# Rhizosphere phage communities drive soil suppressiveness to bacterial wilt disease

**DOI:** 10.1186/s40168-023-01463-8

**Published:** 2023-02-01

**Authors:** Keming Yang, Xiaofang Wang, Rujiao Hou, Chunxia Lu, Zhe Fan, Jingxuan Li, Shuo Wang, Yangchun Xu, Qirong Shen, Ville-Petri Friman, Zhong Wei

**Affiliations:** 1 Joint International Research Laboratory of Soil Health, Jiangsu Provincial Key Lab of Solid Organic Waste Utilization, Jiangsu Collaborative Innovation Center of Solid Organic Wastes, Educational Ministry Engineering Center of Resource-saving fertilizers, Nanjing Agricultural University, Nanjing, 210095 Jiangsu China; 2 Department of Biology, University of York, Wentworth Way, York, YO10 5DD UK; 3 Department of Microbiology, University of Helsinki, 00014 Helsinki, Finland

**Keywords:** Phage community ecology, Viral metagenomics, Rhizosphere virome, Trophic interactions, Bacterial wilt disease, *Ralstonia solanacearum*

## Abstract

**Background:**

Bacterial viruses, phages, play a key role in nutrient turnover and lysis of bacteria in terrestrial ecosystems. While phages are abundant in soils, their effects on plant pathogens and rhizosphere bacterial communities are poorly understood. Here, we used metagenomics and direct experiments to causally test if differences in rhizosphere phage communities could explain variation in soil suppressiveness and bacterial wilt plant disease outcomes by plant-pathogenic *Ralstonia solanacearum* bacterium. Specifically, we tested two hypotheses: (1) that healthy plants are associated with stronger top-down pathogen control by *R. solanacearum*-specific phages (i.e. ‘primary phages’) and (2) that ‘secondary phages’ that target pathogen-inhibiting bacteria play a stronger role in diseased plant rhizosphere microbiomes by indirectly ‘helping’ the pathogen.

**Results:**

Using a repeated sampling of tomato rhizosphere soil in the field, we show that healthy plants are associated with distinct phage communities that contain relatively higher abundances of *R. solanacearum*-specific phages that exert strong top-down pathogen density control. Moreover, ‘secondary phages’ that targeted pathogen-inhibiting bacteria were more abundant in the diseased plant microbiomes. The roles of *R. solanacearum*-specific and ‘secondary phages’ were directly validated in separate greenhouse experiments where we causally show that phages can reduce soil suppressiveness, both directly and indirectly, via top-down control of pathogen densities and by alleviating interference competition between pathogen-inhibiting bacteria and the pathogen.

**Conclusions:**

Together, our findings demonstrate that soil suppressiveness, which is most often attributed to bacteria, could be driven by rhizosphere phage communities that regulate *R. solanacearum* densities and strength of interference competition with pathogen-suppressing bacteria. Rhizosphere phage communities are hence likely to be important in determining bacterial wilt disease outcomes and soil suppressiveness in agricultural fields.

Video Abstract

**Supplementary Information:**

The online version contains supplementary material available at 10.1186/s40168-023-01463-8.

## Background

Plant rhizosphere-associated bacterial communities play a key role in plant health and form the first line of defence against invading pathogens through competition for space and nutrients [1–3]. Invasion-resistant bacterial communities are often characterised by high phylogenetic and functional diversity, which can be linked with high niche occupancy [4] and the presence of ‘inhibitor’ bacteria that can suppress pathogens via the production of antimicrobials or iron-scavenging siderophores [5–7]. While it has been shown that plants can directly select for suppressive microbiota by favouring certain bacterial taxa through ‘host-filtering’ [8], competitive interactions between rhizosphere bacteria can also drive the assembly of suppressive microbiomes [9]. While other groups of microbes, such as fungi or protists, have been associated with suppressive soils [10, 11], the role of the most abundant soil organisms, phages, has been mostly neglected.

Phages are viruses of bacteria and are highly abundant in aquatic and terrestrial ecosystems [12]. They drive the turnover of bacterial biomass through recurrent infections [13] and have several ecosystem-level impacts, affecting soil nitrogen availability [14], carbon cycling [15] and breakdown of pollutants [16]. Previous studies have demonstrated long-term persistence and density fluctuations of phages and their host bacteria in the phytosphere of sugar beets [17, 18], phyllosphere of horse chestnut trees [19–21] and the rhizosphere of tomato plants [22, 23]. Moreover, phages have been shown to drive changes in the diversity and composition of tomato leaf [24] and rhizosphere [22] microbiota and follow temporal changes in bacterial community composition in agricultural soils [25]. Interestingly, in an earlier study, the application of phages also increased the suppressiveness of the rhizosphere microbiota to phytopathogenic *Ralstonia solanacearum* bacterium, likely due to the killing of the pathogen and subsequent release of niche space for antibiotics-producing bacteria [22]. Phages could hence play an important role not only in the composition but also in the functioning of rhizosphere bacterial communities by promoting soil suppressiveness. While it has previously been suggested that healthy and diseased plants might harbour distinct phage communities [26] similar to gut viromes of diseased and healthy humans [27–29], this idea has not been tested experimentally.

**Fig. 1:**
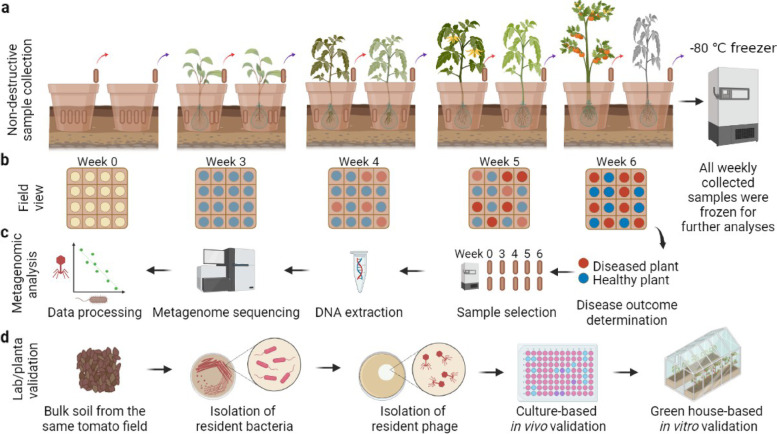
Schematic diagram for experimental design, rhizosphere sample collection and following validation experiments. **a** A semi-natural rhizobox sampling system was embedded in natural tomato field soil to repeatedly sample rhizosphere soil for each plant by removing individual nylon mesh bags inserted in the ‘middle’ layer of the rhizobox, which were immediately stored at − 80 °C. **b** Bacterial wilt disease development was quantified through time within one field plot where red and blue circles denote diseased and healthy plants, respectively. **c** Sample processing flow where four healthy and diseased plants were selected for further temporal analysis and their respective samples from earlier time points were sequenced using shotgun metagenomics. **d** Validation pipeline where bacterial and phage species were isolated from the same field and their effects on the pathogen and bacterial wilt disease outcomes measured in vitro and in planta. All panels were created with BioRender.com

Here, we combined metagenomics and direct experimentation to link rhizosphere phage communities with bacterial wilt disease outcomes in a tomato field—a globally important plant disease of potato, tomato and banana caused by *R. solanacearum* bacterial pathogen [30, 31]. Specifically, we hypothesised that healthy plants could be associated with stronger top-down control by *R. solanacearum*-specific phages (i.e. ‘primary phages’), resulting in lower pathogen densities [22]. Moreover, we wanted to explore how the phage community might indirectly affect *R. solanacearum* densities via the lysis of bacteria that show ‘inhibitory’ interactions with the pathogen [32, 33]. We hence hypothesised that ‘secondary phages’ that target ‘inhibitory’ bacteria could play a relatively more important role in diseased plant rhizosphere microbiomes. To test these hypotheses, we first used metagenomics to track down changes in phage-bacteria population and community dynamics in the rhizosphere microbiomes of healthy and diseased tomatoes infected by the *R. solanacearum* pathogen. The samples were derived from a field experiment, which used a rhizobox system embedded in the field [33] to study the assembly and development of rhizosphere microbiomes using non-destructive sampling of tomato plants (Fig. 1). This approach allowed repeated sampling of the same plant individuals in natural conditions from seedling to fruiting stage and choosing a subset of plants that remained healthy or succumbed to disease for further analysis. To this end, four healthy and four diseased plants were chosen at the end of the experiment, and their past rhizosphere samples collected at weeks 0, 3, 4, 5 and 6 were sequenced to study the underlying differences in bacterial and phage communities using metagenomics (total of 40 metagenomic samples). Further lab and greenhouse experiments were conducted to causally test the direct and indirect effects of *R. solanacearum*-specific and ‘secondary phages’ on the soil suppressiveness by determining their effects on pathogen densities and the interference competition between pathogen-inhibiting bacteria and the pathogen, respectively.

## Results

### Healthy and diseased rhizosphere microbiomes harbour distinct phage communities

We first compared the differences in the overall bacterial and phage communities between healthy and diseased rhizosphere microbiome samples. While the bacterial community alpha diversity varied in time (Shannon index: *F*_4,24_ = 90.4470, *P* < 0.001, Additional file 1: Supplementary Data S1), no significant difference between healthy and diseased plant microbiomes was observed (Additional file 2: Fig. 1 a *P* = 0.17, Additional file 1: Supplementary Data S1). Overall, the bacterial community consisted of 45% of *Proteobacteria* (*n* = 3895), 22% of *Actinobacteria* (*n* = 1952), 20% of *Firmicutes* (*n* = 1711) and 8% *Bacteroidetes* (*n* = 733, Additional file 2: Fig. 1 b). Moreover, bacterial community composition of healthy and diseased plant microbiomes was significantly different and remained dissimilar from the beginning to the end of the experiment (Additional file 2: Fig. 1 c-h).

**Fig. 2:**
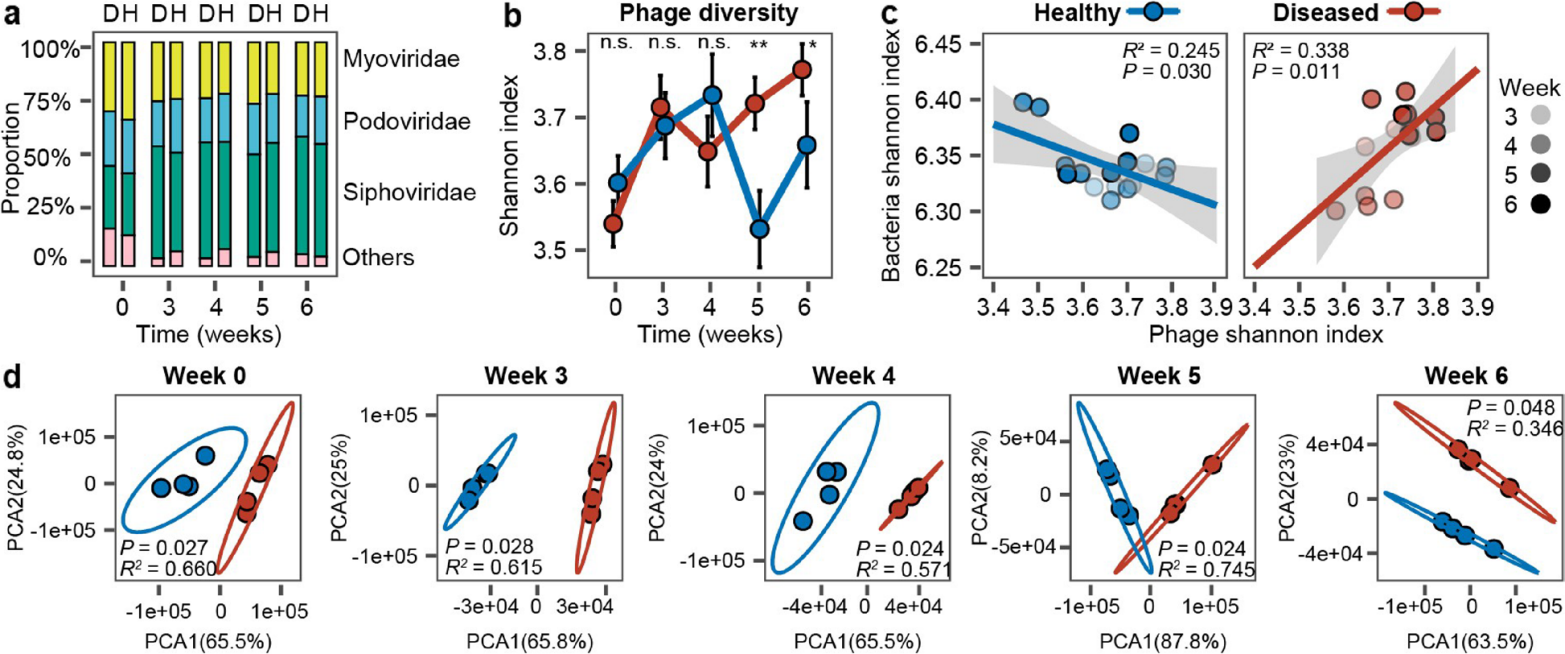
Comparison of phage community composition and diversity between healthy and diseased plant rhizosphere microbiome samples. **a** Comparison of relative phage abundances at the viral family level between healthy (H) and diseased (D) plant rhizosphere microbiome samples (stacked bars show the summed differences of four replicates). **b** Comparison of phage community diversity (Shannon alpha diversity index) between healthy and diseased rhizosphere microbiome samples based on vOTUs. Significances are shown as *P < 0.05, **P < 0.01. n.s., non-significant. One-way ANOVA for each time point (see Additional file 1: Supplementary Data S3 for details). **c** Linear correlations comparing Shannon diversity between bacterial and viral communities in healthy (blue circles) and diseased (red circles) plant rhizosphere microbiomes from weeks 3 to 6. **d** Comparison of viral community composition between healthy (blue circles) and diseased (red circles) plant rhizosphere microbiomes at different sampling time points based on vOTUs (PCA; pairwise comparisons based on PERMANOVA). In all panels, data shows four biological replicates per healthy and diseased plant group

In the case of the viral community, we identified 79 viral operational taxonomic units (vOTUs) with a criterion of 85% coverage and 95% similarity recommended by MIUViG [34] (Additional file 1: Supplementary Data S2). Of all the vOTUs, 26.58% were classified as *Myoviridae* (*n* = 21), 18.99% as *Podoviridae* (*n* = 15), 43.04% as *Siphoviridae* (*n* = 34) and 11.39% as unclassified (*n* = 9, Additional file 1: Supplementary Data S2). Furthermore, we predicted bacterial hosts for all identified vOTUs using VPF-Class [35]: 33.96% of vOTUs were predicted to be linked with *Actinobacteria* (*n* = 18), 3.77% with *Bacteroidetes* (*n* = 2), 3.77% with *Cyanobacteria* (*n* = 2), 13.21% with *Firmicutes* (*n* = 7) and 45.28% with *Proteobacteria* (*n* = 24, Additional file 2: Fig. 2 a). The relative abundance of *Podoviridae* family was generally higher in the healthy plant microbiomes, while *Siphoviridae* family had higher abundances in diseased plant microbiomes when analysed over the whole data (*Podoviridae*: *F*_1,6_ = 17.64, *P* = 0.006; *Siphoviridae*: *F*_1,6_ = 11.63, *P* = 0.014, Fig. 2 a, Additional file 1: Supplementary Data S1). Phage communities were generally more diverse in the diseased plant microbiomes, and this difference was especially clear during weeks 5 and 6 (Shannon alpha diversity index considering both richness and evenness: *F*_1,6_ = 37.15, *P* < 0.001, Fig. 2 b, Additional file 1: Supplementary Data S1, S3). Moreover, phage and bacterial community alpha diversities were negatively correlated in the healthy but positively correlated in the diseased plant microbiomes (Fig. 2 c). Similar to bacteria, phage community composition was consistently different between healthy and diseased plant microbiome samples (Fig. 2 c, d), and while this difference was clearest in initial soil samples, it increased between weeks 3 and 6 (Additional file 2: Fig. 2 b). We also compared the effect of physicochemical soil properties between healthy and diseased samples at the beginning of the experiment. No significant differences were found except for healthy plants having higher levels of total organic carbon (*F*_1,6_ = 9.348, *P* = 0.0223, Additional file 2: Fig. S3). Together, these data suggest that healthy and diseased plants were associated with distinct bacterial and phage rhizosphere communities, while no clear differences in soil physicochemical properties were found.

### Healthy rhizosphere microbiomes contain a relatively higher abundance of *R. solanacearum*-specific phages

**Fig. 3:**
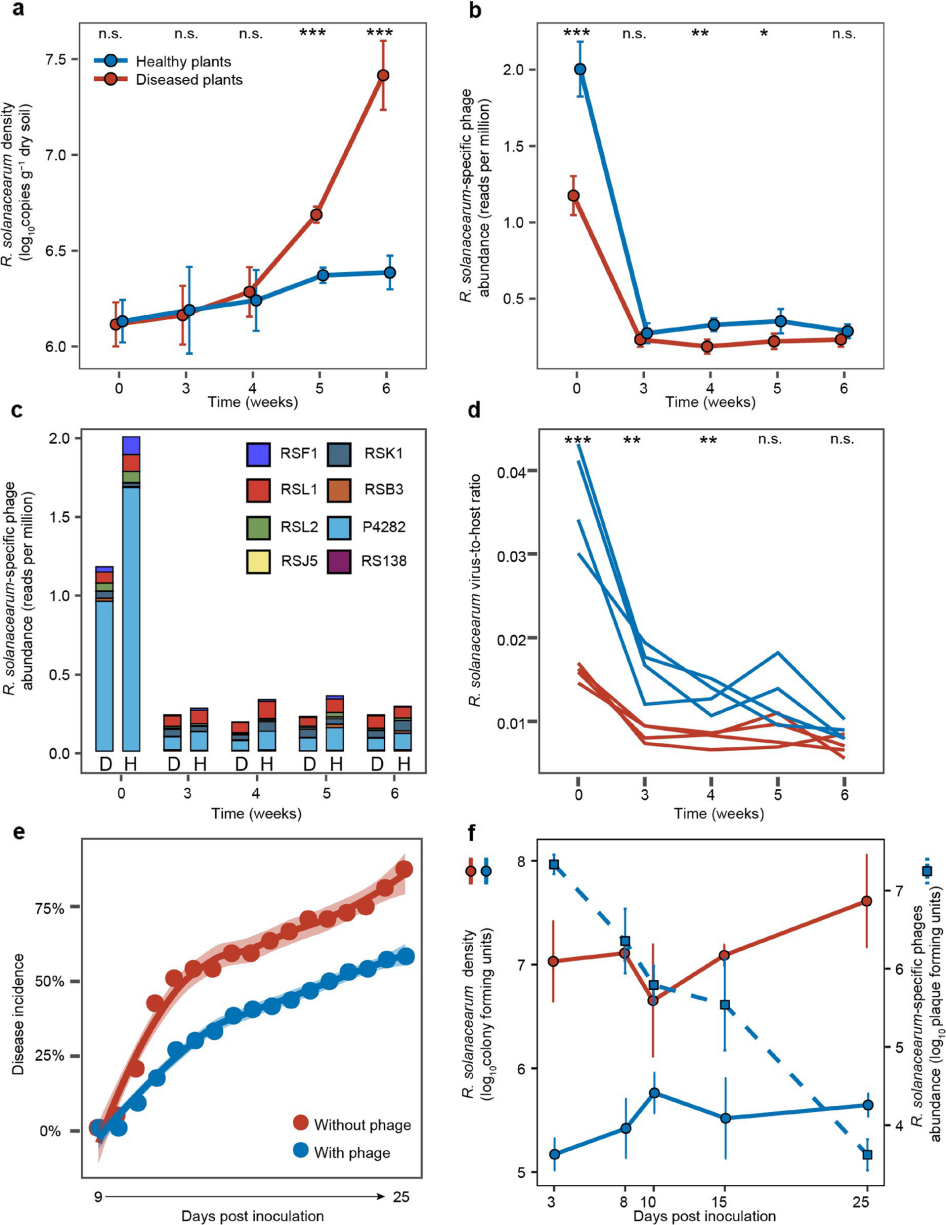
The density dynamics of *R. solanacearum* bacterium and *R. solanacearum*-specific phages in healthy and diseased plant rhizosphere microbiome samples and during a greenhouse validation experiment. **a**, **b** The mean (± SD) density dynamics of *R. solanacearum* bacterium (**a**) and its phages (**b**) in healthy (blue) and diseased (red) plant rhizosphere microbiome samples over time. **c** The average density dynamics of different *R. solanacearum*-specific phage species in healthy (H) and diseased (D) plant rhizosphere microbiome samples over time. **d** Comparison of *R. solanacearum* virus-to-host ratio between healthy (blue line) and diseased (red line) plant samples across time. **e** Bacterial wilt disease development in tomato in the absence (red) and presence (blue) of phages during validation greenhouse experiment. **f** The *R. solanacearum* density dynamics in the absence (red) and presence (blue) of phages (left *Y*-axis); the density of *R. solanacearum*-specific phages is shown on dashed light blue line (right *Y*-axis). In panels **a**, **b**, and **d**, data shows four biological replicates per healthy and diseased plant group, while each line represents an individual biological replicate in **c** (significances are shown as *P < 0.05, **P < 0.01, and ***P < 0.001; n.s., non-significant, one-way ANOVA, see Additional file 1: Supplementary Data S3 for details). In **e** and **f**, data shows four biological replicates per treatment

To investigate if contrasting disease outcomes could potentially be explained by phage-mediated density regulation of the pathogen [22], we quantified *R. solanacearum* densities using quantitative PCR [33] and *R. solanacearum*-specific phage ‘marker’ gene counts as the vOTU approach failed to assemble *R. solanacearum* phage contigs. Specifically, we chose the relatively most abundant gene per phage species per time point as the marker gene to control for bias due to phage species genome size differences. We found that while pathogen densities did not initially differ between healthy and diseased plants, they increased to much higher levels in the diseased plant microbiomes during weeks 5 and 6 (Fig. 3 a, *F*_1,6_ = 24.8, *P* = 0.002, Additional file 1: Supplementary Data S1, S3). In contrast, *R. solanacearum*-specific phages were relatively more abundant in healthy plant microbiomes especially at the beginning of the sampling (*F*_1,6_ = 90.27, *P* < 0.001, Fig. 3 b, Additional file 1: Supplementary Data S1, S3). A total of eight *R. solanacearum*-specific phages could be identified in both healthy and diseased samples (Fig. 3 c), which included three *Myoviridae* phages (RSL2, RSF1 and RSL1), three *Podoviridae* phages (RSK1, RSB3 and RSJ5), one *Siphoviridae* phage (RS138) and one unclassified phage (P4282; Additional file 2: Table S1). Phages P4282 (64.72%) and RSL1 (15.02%) were the most abundant phages overall (Fig. 3 c), while phages RSF1, RSL1 and P4282 showed relatively higher abundances in the healthy plant microbiomes (RSF1: *P* = 0.004, *F*_1,6_ = 18.92; RSL1: *P* = 0.008, *F*_1,6_ = 14.77; P4282: *P* < 0.001, *F*_1,6_ = 121.65, Additional file 2: Fig. S4, Additional file 1: Supplementary Data S1, S3). *R. solanacearum*-specific phage species also showed temporal variation in their peak densities, suggesting that different phages might have been relatively more active during different stages of tomato growth and development (Additional file 2: Fig. S4).

To estimate the potential top-down control of *R. solanacearum* by phages, we compared the phage-bacteria ratio between healthy and diseased samples based on relative abundances derived from the metagenomics dataset (total gene counts for *R. solanacearum* and *R. solanacearum*-specific phages per sample). We found that the phage-bacteria ratio was higher in healthy plant samples over time (*F*_1,6_ = 213.56, *P* < 0.001, Fig. 3 d, Additional file 1: Supplementary Data S1, S3), indicating a higher phage production and potentially stronger top-down density control of the pathogen [36]. To validate this causally, we conducted an additional greenhouse experiment where we compared *R. solanacearum* cell and phage particle densities over time. We found that inoculation of a four-phage combination [22] clearly reduced both disease symptoms (53.13%, Fig. 3 e) and pathogen densities (25.83%, Fig. 3 f) relative to the control treatment without phages. Interestingly, while pathogen density reduction remained stable throughout the experiment, phage densities declined towards the end of the experiment similar to what was observed in the metagenomic field experiment data (Fig. 3 b, f). Together, these results show that healthy tomato microbiomes were associated with a relatively higher abundance of pathogen-specific phages, which likely exerted relatively stronger top-down density regulation on *R. solanacearum*.

### Phage communities can indirectly drive bacterial wilt disease outcomes by alleviating competition between the pathogen and ‘inhibitor bacteria’

#### Indirect evidence based on correlations and co-occurrence analysis

As *R. solanacearum* invasion success is heavily modulated by the presence of antagonistic and facilitative rhizosphere bacteria [4, 6, 32, 33, 37, 38], we next tested if the phage community could have indirectly affected pathogen densities by infecting bacteria that are positively (‘facilitator bacteria’) or negatively (‘inhibitor bacteria’) associated with the *R. solanacearum* (see the ‘Materials and methods’ section). To this end, we compared the non-redundant bacterial and phage gene counts between the healthy and diseased plant samples across time. A total of 606 phage species and 568 phage-bacteria pairs could be established at the species level using Virus-Host DB [39] (Additional file 1: Supplementary Data S4). To focus on potential top-down density regulation via lysis, only phage-bacteria associations predicted to be lytic based on phageAI [40] were included in these analyses. The *R. solanacearum*-specific phages showed significant negative correlations with the pathogen in both healthy and diseased plant microbiome samples (SparCC *P*-value < 0.05, Additional file 1: Supplementary Data S5) and were classified as ‘primary phages’. We then established associations between *R. solanacearum* and inhibitor (SparCC *P*-value < 0.05, cov < 0, Additional file 1: Supplementary Data S5) and facilitator bacteria (SparCC *P*-value < 0.05, cov > 0, Additional file 1: Supplementary Data S5; also designated as direct ‘primary’ effects) and finally determined correlations between ‘inhibitor’ and ‘facilitator’ bacteria and their predicted phages (SparCC *P*-value < 0.05, Additional file 1: Supplementary Data S5; classified as ‘secondary effects’).

**Fig. 4:**
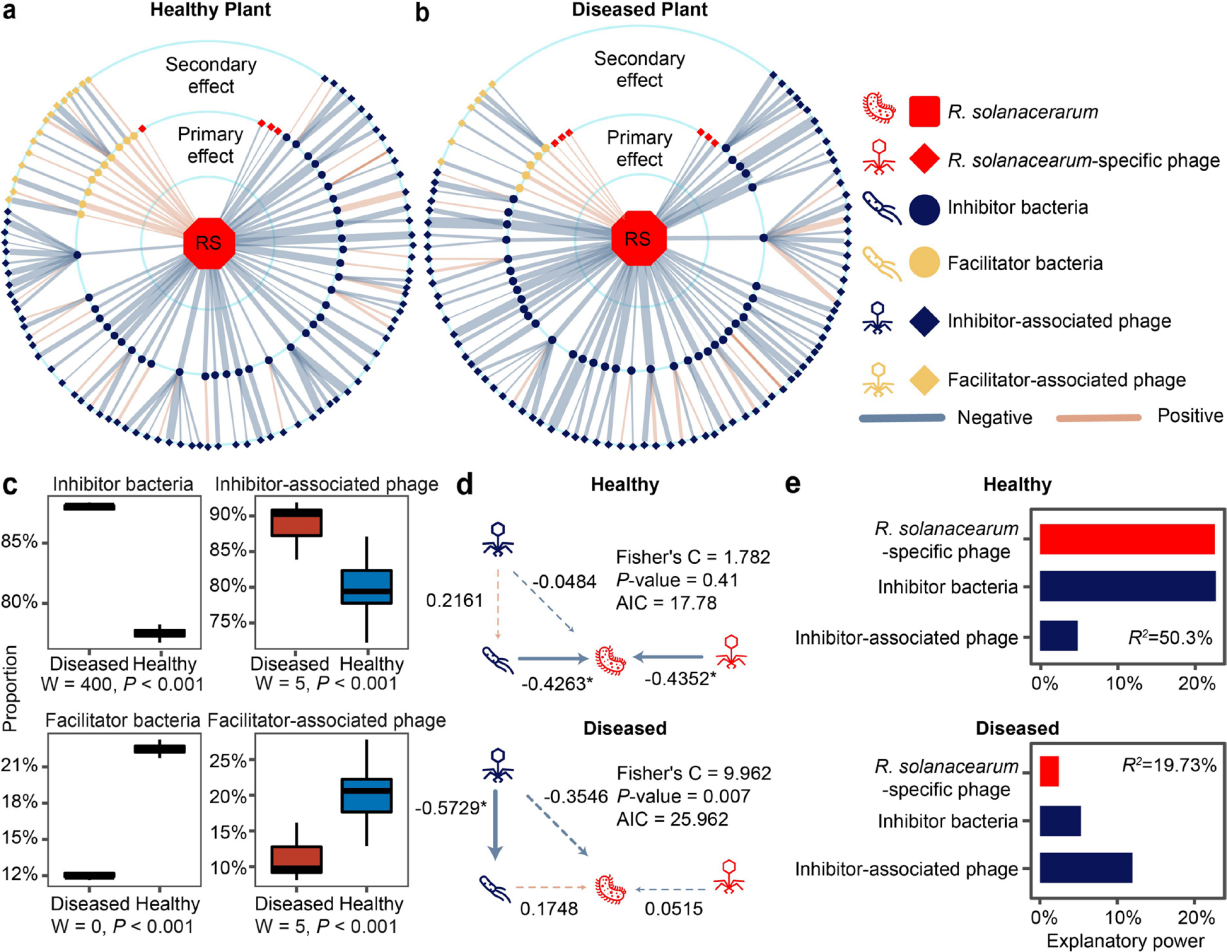
Community co-occurrence model showing correlations between *R. solanacearum*, bacteria and phages in healthy and diseased plant rhizosphere microbiome samples. **a**, **b** Radial co-occurrence networks showing significant correlations between *R. solanacearum* (RS; middle), inhibitor (blue circles) and facilitator (beige circles) bacteria and ‘*R. solanacearum*-specific’ phages (red diamond) and ‘inhibitor-associated’ (blue diamonds) or ‘facilitator-associated’ (beige diamonds) phages. Light blue and light brown lines denote negative and positive correlations, respectively, while line thickness represents the absolute sparcc-cov-values (Additional file 1: Supplementary Data S5). **c** Comparison of the proportion of different ‘functional groups’ between healthy (blue) and diseased (red) rhizosphere microbiome samples (*n* = 4, statistical analysis based on Wilcoxon non-parametric test). **d** Structural equation model illustrating significant links between *R. solanacearum*, ‘inhibitor bacteria’, ‘inhibitor-associated phages’ and ‘*R. solanacearum* phages’ in healthy and diseased plant rhizosphere samples. **e** The partition of explanatory power of linear models predicting *R. solanacearum* densities with ‘*R. solanacearum* phages’, ‘inhibitor bacteria’ and ‘inhibitor-associated phages’ in healthy and diseased plant rhizosphere microbiome samples (*R*^2^ shows the total variance explained by the linear models)

When visualised as networks, where line colours and thickness denote the direction and strength of correlations (Fig. 4 a, b), only a few structural differences were found: healthy and diseased plant co-occurrence networks contained 139 and 137 nodes and 140 and 138 edges, respectively, and had the same connectivity of 1 (Additional file 2: Table S2). However, the relative abundances of different functional groups within each network were different. Specifically, the diseased plant microbiome networks contained a higher proportion of ‘inhibitor’ bacteria (88.37% vs 78.26%, *P* < 0.001, Fig. 4 a–c, Additional file 2: Fig. S4a-b) and ‘inhibitor-associated’ phages (92.05% vs 83.15%, *P* < 0.001, Fig. 4 a–c, Additional file 2: Fig. S4a-b), while healthy plant networks contained a higher proportion of facilitator bacteria (21.74% vs 11.63%, *P* < 0.001, Fig. 4 a–c, Additional file 2: Fig. S5a-b) and ‘facilitator-associated’ phages (16.85% vs 7.95%, *P* < 0.001, Fig. 4 a–c, Additional file 2: Fig. S5a-b).

**Fig. 5:**
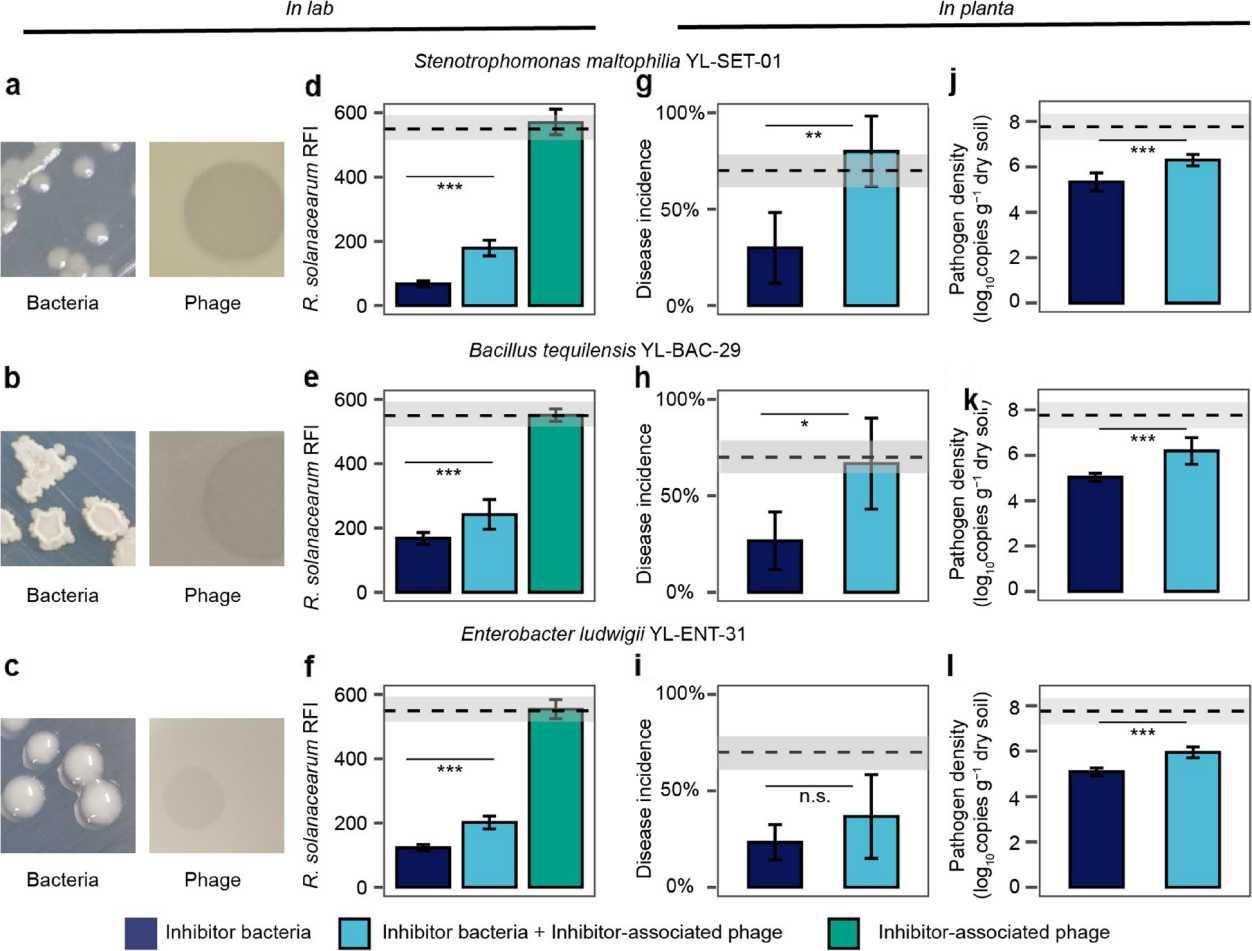
Experimental validation of the effects of ‘secondary phages’ on pathogen abundances and bacterial wilt disease incidence. **a**–**c** Examples of colony and plaque morphologies of three ‘inhibitor bacteria’ and ‘inhibitor-associated phages’. **d**–**f** The effect of three ‘inhibitor bacteria’ and ‘inhibitor-associated phages’ on *R. solanacearum* density (red fluorescence intensity (RFI)) in all possible combinations, *n* = 8 for all treatments. **g**–**l** The effect of three ‘inhibitor bacteria’ on bacterial wilt disease incidence (**g**–**i**, *n* = 5 for all treatments) and pathogen density (**j**–**l**, *n* = 6 for all treatments) when applied alone or co-inoculated with ‘inhibitor-associated phages’ during tomato greenhouse experiments. In **d**–**l**, dashed lines and shading indicate the mean and standard deviation of pathogen-only control treatments, respectively, and statistical significances between treatments were determined by one-way ANOVA where *P < 0.05, **P < 0.01, ***P < 0.001. n.s., non-significant (see Additional file 2: Tables S8–S11 for details; all error bars show standard deviation)

To analyse the relative importance of these ‘primary’ and ‘secondary’ effects on *R. solanacearum* abundances, two-step piecewise structural equation (SEM) models were constructed. Based on model fitting, the Shannon diversity index was used due to the best fit (high *P*-value and low Akaike Information Criterion; Additional file 2: Fig. S5c–e, Table S3) and because it captured both diversity and evenness effects of species correlations within each functional group. At this stage, the ‘facilitator bacteria’ and ‘facilitator-associated phages’ groups were removed from the final models due to non-significant effects with *R. solanacearum* (Additional file 2: Fig. S5c, Table S4). According to the final SEM models, *R. solanacearum*-specific phages and ‘inhibitor bacteria’ had negative associations with pathogen abundances only in the healthy plant microbiome (Fig. 5 d, Additional file 2: Table S5). In contrast, the negative effect of ‘inhibitor-associated phages’ was only significant in the diseased plant microbiome, indicative of relatively stronger top-down regulation on ‘inhibitor bacteria’ (Fig. 5 d; also supported by a separate linear model: Additional file 2: Table S6). In support of this, the relative abundance of bacterial genes associated with secondary metabolism and antibiosis [33] was much higher in healthy compared to diseased microbiomes samples at week 6 (nonribosomal peptides: COG1020, *P* = 0.008, *F*_1,6_ = 15.1580; polyketide synthase: COG3321, *P* = 0.03, *F*_1,6_ = 7.8523; amino acid adenylation: NOG01415, *P* = 0.003, *F*_1,6_ = 20.6590, one-way ANOVA, Additional file 1: Supplementary Data S3, Additional file 2: Fig. S6). The relative importance of ‘primary’ and ‘secondary’ effects on pathogen abundances was also compared based on the explanatory power of constructed linear models. In line with the SEM, both the ‘inhibitor bacteria’ and ‘primary phages’ had a relatively higher explanatory power in healthy plant microbiome (Fig. 4 e; Additional file 2: Table S7), while ‘inhibitor-associated phages’ explained relatively more of the pathogen density variation in diseased plant microbiome. Together, these results suggest that pathogen densities might have been regulated by direct and indirect interactions by bacterial and phage communities.

#### Direct evidence based on experiments conducted in the lab and in planta

To experimentally validate the importance of ‘secondary phages’ on bacterial wilt disease severity, we isolated three ‘inhibitor bacteria’ and their associated phages from the field soil where the original experiment was conducted (Qilin, Nanjing, China; see the ‘Materials and methods’ section). Based on 16S rRNA Sanger sequencing, isolated bacteria were classified as *Stenotrophomonas maltophilia* YL-Ste-01, *Bacillus tequilensis* YL-Bac-29 and *Enterobacter ludwigii* YL-Ent-31 (Additional file 2: Fig. S7a-c). Of these strains, *S. maltophilia* was identified as an ‘inhibitor bacterium’ in our metagenomic dataset, while *B. tequilensis* was closely related to another ‘inhibitor bacteria’, *B. subtilis*, identified in the healthy plant microbiome metagenomes (99% similarity). All isolated phages formed clear plaques on soft agar overlays, suggesting that they were lytic (Fig. 5 a–c) and likely belonged to *Siphoviridae* and *Myoviridae* families based on TEM images (Additional file 2: Fig. S7d-f). Phages were also specific to each ‘inhibitor bacteria’ species that could not infect *R. solanacearum* QL-Rs1115 type strain [41] (Fig. 5 d–f, Additional file 2: Fig. S8, Tables S8-S9). We first used simple lab assays to show that all ‘inhibitor bacteria’ had highly negative effects on the pathogen densities (*S. maltophilia*: *P* < 0.001, *F*_1,14_ = 1652, *B. tequilensis*: *P* < 0.001, *F*_1,14_ = 875.9, *E. ludwigii*: *P* < 0.001, *F*_1,14_ = 1291, one-way ANOVA, Fig. 5 d–f, Additional file 2: Table S9). Crucially, pathogen suppression by the ‘inhibitor bacteria’ was clearly reduced in the presence of ‘inhibitor-associated phages’ with all species (*S. maltophilia*: *P* < 0.001, *F*_1,14_ = 145.4, *B. tequilensis*: *P* < 0.001, *F*_1,14_ = 17.8, *E. ludwigii*: *P* < 0.001, *F*_1,14_ = 99.9, one-way ANOVA, Fig. 5 d–f, Additional file 2: Table S9).

To validate these findings in planta, we conducted a greenhouse experiment with tomatoes using a sterile planting substrate and the same experimental treatments as in the lab assays. In addition to quantifying pathogen densities using qPCR, we also analysed the effects of ‘inhibitor bacteria’ and ‘inhibitor-associated phages’ on bacterial wilt disease incidence. *R. solanacearum*-mono control treatment showed approximately 70% disease incidence by the end of the experiment. In contrast, tomato plants inoculated with both the pathogen and ‘inhibitor bacteria’ showed clear percentage reductions in disease symptoms (*S. maltophilia*, 57.14%; *B. tequilensis*, 61.90%; *E. ludwigii*, 66.67%, Fig. 5 g–i, Additional file 2: Fig. S9, Table S10) and pathogen densities (*S. maltophilia*, 31.27%; *B. tequilensis*, 35.18%; *E. ludwigii*, 34.43%, Fig. 5 j–l, Additional file 2: Table S11). These effects however vanished in the presence of ‘inhibitor-associated phages’, leading to even higher disease symptoms compared to *R. solanacearum*-mono control treatment with *S. maltophilia* and *B. tequilensis* (166.67% and 150.09% percentage increases, respectively), while a similar but statistically non-significant trend was observed with *E. ludwigii* (57.51% increase, one-way ANOVA, Fig. 5 g–i, Additional file 2: Fig. S9, Table S10). In line with the disease incidence data, pathogen densities increased in the presence of ‘inhibitor-associated phages’ (*S. maltophilia*, 18.04%; *B. tequilensis*, 23.02%; *E. ludwigii*, 16.75%, Fig. 5 j–l, Additional file 2: Table S11). Together, this data demonstrates that ‘secondary phages’ can indirectly promote bacterial wilt disease by alleviating competition between the pathogen and ‘inhibitor bacteria’.

## Discussion

While it has been suggested that differences in phage communities could be associated with bacterial plant disease [26], experimental evidence has been lacking. Here, we combined metagenomics and direct experimentation to test two hypotheses: (1) that healthy plants are associated with stronger top-down control by *R. solanacearum*-specific phages (i.e. ‘primary phages’) and (2) that ‘secondary phages’ that target pathogen-inhibiting bacteria could play a relatively more important role in diseased plant rhizosphere microbiomes. We found support for both hypotheses. First, healthy plant phage communities contained a relatively higher total abundance of ‘primary phages’ that could directly infect the *R. solanacearum* plant pathogenic bacterium. Three phage species were relatively more abundant in healthy plant microbiomes. Of these, RSL1 is a jumbo phage, which belongs to the *Myoviridae* family and has previously been shown to be highly effective at controlling *R. solanacearum* densities and bacterial wilt disease in the lab and greenhouse conditions [42]. Similarly, phages RSF1 and P4282 that had relatively higher abundances in the healthy plant rhizosphere have previously been shown to be capable of inhibiting bacterial wilt progression in tomato and tobacco, respectively [43, 44], while phage RSK1 has high similarity with previously characterised Chinese phages capable at infecting *R. solanacearum* [22]. We have also previously isolated *R. solanacearum*-specific phages from the same and three other tomato fields and demonstrated that they are together highly effective at controlling *R. solanacearum* densities and bacterial wilt disease incidence both in greenhouse and field experiments [22, 23]. To validate these metagenomic results, we used a phage cocktail consisting of these four phages [22] to causally show that phages can keep the pathogen densities and bacterial wilt disease incidence in check. Interestingly, we also observed a clear decrease in total phage densities in time without a concomitant decrease in *R. solanacearum* abundances similar to metagenomic data. One reason for this discrepancy could be that phage-bacteria population dynamics are much faster than our sampling interval of 1 week (or few days in the validation experiment), which could explain why phage-bacteria dynamics appeared to be uncoupled. Furthermore, it is possible that bacteria could have evolved phage resistance as observed previously [22, 23], which could have decoupled population dynamics, leading to a reduction in phage but not in bacterial abundances due to them being resistant. Together, these findings suggest that *R. solanacearum*-specific phages exerted relatively stronger top-down regulation in healthy tomato rhizosphere microbiomes, similar to the phage-mediated top-down control of bacteria in aquatic [45, 46], plant phyllosphere [24] and other terrestrial environments [25, 47].

We also found that phage communities had an important indirect role in bacterial wilt disease progression by affecting the strength of competitive interactions between pathogen-inhibiting bacteria and *R. solanacearum*. Several inhibitory bacteria have been linked to a reduction in bacterial wilt disease incidence [4, 6, 37], and we have previously shown that *Bacillus* and *Pseudomona*s bacteria isolated from the same field showed a higher *R. solanacearum* inhibition in vitro when they originated from healthy versus diseased plant rhizosphere microbiomes [33]. In line with this, also the isolated *B. tequilensis* bacterium showed a clear *R. solanacearum* growth inhibition both in vitro and in planta, in addition to two other inhibitory bacteria, *S. maltophilia* and *E. ludwigii.* While the mechanism of inhibition remains unclear, we found that healthy plant microbiomes contained a relatively higher abundance of bacterial genes associated with secondary metabolism and antibiosis [33]. In combination with inhibition assay data, pathogen growth suppression via secretion of antimicrobial compounds is hence the most plausible explanation for our results, even though we cannot exclude the potential importance of other factors, such as iron-scavenging siderophores [6] or resource competition [4]. Interestingly, we have previously demonstrated that *R. solanacearum*-specific phages can increase the relative abundance of inhibitory bacteria in the rhizosphere [22] and that application of *B. amyloliquefaciens* inhibitory bacterium together with *R. solanacearum*-specific phage can lead to increased pathogen suppression [23]. It is hence possible that ‘primary’ and ‘secondary’ phages could have interacted synergistically via the direct killing of the pathogen and by magnifying the suppressive effect of inhibitory bacteria. It is however important to note that ‘secondary’ phages could also have negative effects on plant health if they target plant growth-promoting bacteria as observed previously [48]. Moreover, it has been recently shown that *R. solanacearum* can benefit from the presence of ‘facilitative’ bacteria during infections [32, 37]. While we also identified potential facilitative associations between certain bacteria and *R. solanacearum*, their relative importance in explaining bacterial wilt disease outcomes was much smaller compared to ‘inhibitory bacteria’.

Further compositional differences were also observed between healthy and diseased plant rhizosphere phage communities, and overall, phage diversity was higher in diseased plant microbiomes, especially with the last two sampling points. This diversity difference was unlikely explained by the relatively higher bacterial diversity, which did not differ between healthy and diseased samples at these sampling time points. Also, differences in phage community composition and diversity were unlikely explained by differences in soil physicochemical properties, such as pH [49], as only the total organic matter content was found to be initially higher in the healthy plant rhizosphere. Likely more frequent temporal sampling and quantification of biotic and abiotic soil properties are required to better understand the fluctuations in rhizosphere microbiome diversity and composition in time. Together, our findings demonstrate that soil suppressiveness, which is most often attributed to bacteria, could be determined by the top-down density regulation by both antagonistic bacteria and the phage community.

Our findings bear similarities with gut microbiome research in humans. For example, research on type 2 diabetes suggests that gut phages show a strong connection with human health via interactions with a particular host bacterial taxa [50], while another study on inflammatory bowel disease observed disease-specific changes in virome (mostly phage community) but not with bacterial communities [51]. Recent direct experimental evidence further suggests that beneficial effects of phages can be transferred from donor to hosts to increase protection against necrotising enterocolitis in piglets [52] and to improve memory in flies, mice and humans [53]. Similar to this study, we previously demonstrated that the suppressive effect of a healthy tomato rhizosphere microbiome could be transferred to the next plant generation via soil transplantation [33]. While this effect was attributed to the presence of suppressive bacteria, our current results suggest that it could also have been driven by the difference in the rhizosphere phage community. In addition to phages, predatory protists have recently been linked to healthy plants and lower bacterial wilt disease incidence [54] and studies focusing on microbial interactions across multi-level trophic networks are hence required to better understand the key drivers behind suppressive soils at the community level. We also must note that we likely undersampled the viral diversity in our field experiment due to the small volume of the sampled filter bags and lack of phage enriching. Hence, we likely missed some rare viral taxa in our analysis. However, the fact that we could still discern clear differences in viral community composition between healthy and diseased plants suggests that we could capture the representative variation of more common viral taxa. Resuspending and enriching phages in soil samples before isolation and sequencing of phage metagenome libraries can be used to increase the viral taxonomic resolution to assess the role of rarer viral taxa and to assemble larger viral contigs for the identification of viral auxiliary metabolism genes [16, 25, 55]. This will also enable clearer identification of prophages and lysogenic phages from bacterial metagenomes to assess their role in bacteria-phage interactions in rhizosphere microbiomes.

## Conclusion

Our study demonstrates that soil suppressiveness, which is most often attributed to bacteria, could be driven by rhizosphere phage communities. First, we find that healthy plants are associated with stronger top-down control by *R. solanacearum*-specific phages. Second, we show that ‘secondary phages’ have a stronger effect in the diseased plant rhizosphere, alleviating interference competition between the *R. solanacearum* and pathogen-suppressing bacteria. In the future, it will be important to consider the potential role of bacteria-phage evolution and coevolution for these interactions, which have previously been shown to take place within one tomato growth cycle in the rhizosphere [22, 23] or over seasons in the phyllosphere of horse chestnut trees [19–21]. This could be achieved in longer-term selection experiments that take advantage of the ‘mark-recapture’ approach to retrieve back the evolved focal pathogen species for fitness assays and genome resequencing [56]. From the applied perspective, our results suggest that soil suppressiveness is an emergent, microbiome-level property, determined by both bacterial and phage interactions. Phage-mediated biocontrol of plant bacterial diseases could thus be achieved by targeting the pathogen or surrounding microbiota to indirectly constrain pathogen invasions.

## Materials and methods

### Experimental design and collection of soil samples

The experimental design, collection of samples and measurements for soil physicochemical properties have been described in detail in a previous publication [33] where we studied the role of initial bacterial community composition for the dynamics and outcomes of bacterial wilt disease. Briefly, we used a semi-natural rhizobox sampling system embedded in natural tomato field soil, allowing repeated sampling of the same tomato plants during one crop season by removing individual nylon mesh bags inserted in the ‘middle’ layer of the rhizobox (for more details of the system, please see [33]). Each studied tomato plant (*Solanum lycopersicum* cv. ‘Jipin’) was grown individually in its own rhizobox using the local soil from the tomato field in Qilin, Nanjing, China. The first soil samples were collected from the field soil. The same soil samples were subsequently used for setting up rhizoboxes with tomatoes, which were transplanted to the field at the same locations where the original samples were collected (three blocks; 16 plants per block; each plant located 30 cm from each other). This allowed us to compare the initial bulk soils with samples collected from the rhizoboxes at weeks 3, 4, 5 and 6 after the transplantation of tomatoes to the field. At every sampling, four individual nylon bags per plant were collected, and their contents homogenised and pooled together for the microbiome analysis (immediately stored at − 80 °C for further analysis; see below). The nylon bags were located in close proximity to the plant roots and could hence be considered as rhizosphere communities affected by tomato root exudates (Fig. 1 a). At the end of the experiment, we randomly chose four plants that remained healthy throughout the experiment and four plants that succumbed to bacterial wilt disease. The five past rhizosphere samples of these selected plants were then processed (a total of 40 samples) to compare the initial and temporal changes in microbiome assembly [33] using metagenomic sequencing (Fig. 1 b, c).

### Sample preparation, Illumina Hiseq sequencing and identification of bacteria and phages in metagenomic samples

The total microbial DNA from all 40 samples was extracted using the E.Z.N.A.® stool DNA Kit (Omega Bio-Tek, Norcross, GA, USA) according to the manufacturer’s protocols. DNA quality was tested by NanoDrop 2000 Spectrophotometer (Thermo Scientific, DE, USA, Additional file 1: Supplementary Data S6). Metagenomic sequencing was performed using Illumina HiSeq X instrument with pair-end 150 bp (PE150) mode at Shanghai Biozeron Biological Technology Co. Ltd. (Shanghai, China) [33]. We next followed up a standard library preparation protocol without enrichment [57] where 1 μg of genomic DNA was sheared for each sample using Covaris S220 Focused-ultrasonicator (Woburn, MA, USA). Sequencing libraries were then prepared with a fragment length of approximately 450 bp (Additional file 1: Supplementary Data S7). Trimmomatic [58] was used to remove adaptors, contaminants and low-quality reads (version: 0.36, settings: ILLUMINACLIP:adapters.fa:2:30:10 SLIDINGWINDOW:4:15 MINLEN:75, Additional file 1: Supplementary Data S8). Clean sequence reads were assembled into a set of contigs for each sample using MegaHit (version: 1.1.1-2-g02102e1, settings: --min-contig-len 500) [59] (Additional file 1: Supplementary Data S9).

As vOTU contig sizes ranged from 5 to 110.2 kb with an average size of 19.7 kb in our dataset, the VirSorter2 pipeline was chosen for the analysis as it works well with relatively longer contigs (> 10 kb) [60]. We first predicted viral contigs by VirSorter2 (version: 2.2.3, contig length > 5 kb, score > 0.9, *p*-value < 0.05, hallmark > 2) and clustered into 79 viral operational taxonomic units (vOTUs) with a criterion of 85% coverage and 95% similarity. We next calculated the read counts of each vOTU and normalised their abundances following transcripts per million (TPM) method [61], which corrects data for variation in contig length and mapped reads per sample. Finally, we used vpf-class [35] (version: 15 July 2022) to annotate the viral taxa (confidence score > 0.36) and predict their host bacteria (confidence score > 0.5 and membership ratio > 0.3).

We also created a non-redundant gene catalogue based on all sequence data for analysing community co-occurrence patterns between healthy and diseased plant samples. A total of 42,959,757 open reading frames (ORFs) were predicted using Prodigal (v2.6.3, default settings) [62] using all assembled contigs (Additional file 1: Supplementary Data S10). These ORFs were then used to create a non-redundant gene catalogue, which included 12,800,400 unique genes after clustering using CD-HIT [63] (version: 4.8.1, 95% identity, 90% coverage). All the genes included in the catalogue were annotated by their taxonomy using the best hits in NCBI RefSeq NR database (diamond, version: 0.9.22.123, setting: blastp --evalue 0.00001, release number: 90) and functions by eggNOG database [64] (version: v4.5, default settings). At the end, our metagenomic sequences contained a total of 12,847 unique phage and 6,977,753 bacterial genes. Based on BLASTp, we could identify a total of 14,651 microorganisms, which included 11,732 bacteria, 1311 eukaryotes, 907 archaea and 701 viruses at the species level (*e*-value < 0.00001). SOAPaligner [65] (version: r242, default settings) was then used to map and standardise clean read counts in total reads back to the total non-redundant gene catalogue counts per sample based on a 95% sequence identity threshold to calculate each annotated gene abundances, which were corrected for variation in gene length [66]. The abundances of genes annotated to the same taxonomy were then summed up as the taxonomic abundances per sample.

Of the viral sequence data, a total of 606 phages could be assigned to *Caudovirales*, *Microviridae*, *Tectiviridae*, and ‘unclassified’ phage groups. Furthermore, to determine the proportion of prophages of all phage sequences, all identified phage genes were run through the PHASTER [67] prophage protein database (version: December 22, 2020), resulting in 320 prophage hits (identity ≥ 90%, alignment length ≥ 30 bp, *e*-value < 0.00001).

### Analysis of metagenomic data

#### Comparing the viral and bacterial community composition between healthy and diseased tomato soil samples

With viral community analysis, vOTUs were combined at the family level (*Myoviridae*, *Podoviridae*, *Siphoviridae* and ‘others’) to calculate relative (percentage) abundances, which were compared between healthy and diseased microbiomes over time. The raw vOTU dataset was used to compare both alpha (Shannon diversity index) and beta (PCA) diversity between microbiome samples. This dataset was also used to calculate the Bray-Curtis dissimilarity distance between healthy and diseased plant microbiome composition at each time point. Finally, vOTUs that were predicted to infect the same host bacteria were pooled together and visualised using the Krona plot (version: v2.8) [68].

With bacterial community analysis, a non-redundant gene catalogue dataset was used for community composition and diversity analyses. Species-level data was used to compare both alpha (Shannon diversity index) and beta (PCA) diversities between microbiome samples and to calculate the Bray-Curtis dissimilarity distance between healthy and diseased plant microbiomes at each time point. We also combined bacterial species at the phylum level to compare the relative (percentage) abundances between healthy and diseased microbiomes.

#### Quantifying the abundances of R. solanacearum and R. solanacearum-specific phages during the experiment

*R. solanacearum* pathogen abundances were determined using qPCR from the same soil DNA samples that were also used for metagenomic sequencing [33]. In the case of *R. solanacearum* phages, we used a non-redundant gene catalogue approach where we used the most abundant gene for each phage as the metagenomic ‘marker gene’ due to relatively short *R. solanacearum* phage contig length (on average 724 bp, which is too short for vOTU approach). To attain relative phage species abundances relative to all detected *R. solanacearum*-specific phages, read counts of these genes were normalised with marker gene sizes and the number of total clean reads per each sample to prevent bias due to different marker genes used during different sampling weeks [66] (the marker genes per phage species at different weeks and sample replicates are listed in Additional file 1: Supplementary Data S11). Phage species abundances were further summed up as the total *R. solanacearum*-specific phage abundances. Moreover, *R. solanacearum* densities were also estimated based on the metagenomic data using the gene catalogue approach when comparing the virus-to-host ratio with *R. solanacearum*-specific phages.

#### Construction of phage-bacterium co-occurrence model based on SparCC correlations

First, to explore the potential competitive and facilitative interactions between *R. solanacearum* and the members of rhizosphere bacteria, we established links between the pathogen and all identified rhizosphere bacteria and their predicted phages using Virus-Host DB [39] pipeline (Release202) and SparCC algorithm [69], which is capable of estimating correlation values from compositional data (Additional file 1: Supplementary Data S5). To analyse the correlations between bacteria, we used relative bacterial abundance data (%) to normalise between sample variation [70]. To analyse bacteria-phage correlations, we used bacterial and phage abundance data normalised with the total number of gene catalogue counts per sample. The lifecycle of each phage was predicted using phageAI [40] (version: June 2021, default settings, Additional file 1: Supplementary Data S4), and it was found that 37.0% (*n* = 210) of phages were classified as temperate and 42.6% (*n* = 242) lytic, while 20.4% (*n* = 116) of phages could not be clearly classified to either lifestyle. To focus on potential top-down density regulation via lysis, only predicted lytic phage-bacteria associations were included in downstream analysis.

Significant correlations (two-sided pseudo-*P*-value < 0.05 with 999 permutations) between *R. solanacearum* and lytic *R. solanacearum-*specific phages were classified as ‘primary phage effects’, while significant correlations between *R. solanacearum* and other bacteria were determined as ‘primary bacterial effects’. Significant correlations between non-*R. solanacearum-*specific phages and other bacteria were determined as ‘secondary phage effects’. The significance of these three ‘functional groups’ on *R. solanacearum* abundances was explored using networks, which were visualised in Cytoscape [71] (v3.6.0) and the proportion of significant correlations per each functional group was compared between healthy and diseased correlation networks. Network properties were calculated by R package ‘igraph’ [72]. Furthermore, we calculated the Shannon index, Simpson index and average abundance per sample based on the species abundance matrix for each functional group based on SparCC correlations. We then conducted two linear piecewise structural equation models (PSEM) [73] to explore the ‘primary phage effects’, ‘primary bacterial effects’ and ‘secondary phage effects’ on *R. solanacearum* abundances in healthy and diseased rhizosphere microbiome samples. After model fitting, the Shannon diversity index was chosen for further analysis due to best fit (high *P*-value and low Akaike Information Criterion; Additional file 2: Fig. S4c-e, Table S3). Furthermore, ‘facilitator bacteria’ and ‘facilitator-associated phages’ were removed from the final models due to non-significant effects (Additional file 2: Fig. S4c, Table S4). Finally, we conducted an independent linear model to explain the effect of ‘inhibitor-associated phage’ on ‘inhibitor-bacteria’ directly. In addition, these linear models were used to compare the explanatory power of each ‘functional group’ on *R. solanacearum* abundances in healthy and diseased plants using the ‘relaimpo’ package [74].

### Culture-based validation testing the effect of *R. solanacearum*-specific phages on bacterial wilt disease incidence and top-down density control of *R. solanacearum*

We conducted an additional greenhouse experiment to directly validate the effects of *R. solanacearum*-specific phages on the pathogen density and bacterial wilt disease incidence, while also temporally tracking changes in phage abundances. At the three-leaf stage, tomato plants (*Lycopersicon esculentum*, cultivar ‘Micro-Tom’) were transplanted into 6-cell trays with 50 g of thoroughly mixed topsoil per cell, which was collected from the same tomato field as in the rhizobox experiment (Qilin, Nanjing, China). After 1 week from transplantation, plant roots were inoculated with *R. solanacearum* QL-Rs1115 type strain (also isolated from Qilin, Nanjing) at a final concentration of 10^6^~10^7^ CFU g^−1^ soil. Two days later, a phage cocktail consisting of four *R. solanacearum*-specific phages at equal frequencies of 25% (NJ-P3, NB-P21, NC-P34, NN-P42; previously described in [22]) was applied to plant roots with a final density of approximately 10^6^~10^7^ PFU g^−1^ soil. One of these phages (NJ-P3) was also isolated from Qilin, Nanjing, while others were isolated from tomato fields in Ningbo, Nanchang and Nanning [22]. Four biological replicates were used per ‘no-phage’ and ‘phage’ treatments, and one replicate consisted of 6 plants grown on one plant tray (48 plants in total). Plants were grown in a greenhouse with a natural temperature variation ranging between 25 and 35 °C for a total of 25 days post-infection, and trays were randomly rearranged every 3 days. Disease incidence was recorded every day post-pathogen using disease index [22].

To quantify the changes in pathogen and total phage densities, we collected rhizosphere soil samples 3, 8, 10, 15 and 25 days post-pathogen inoculation. For density calculations, 1 g of rhizosphere soil was mixed with 9 mL of sterile water to create a soil wash. The *R. solanacearum* densities were detected with a serial dilution method on SMSA medium [75] based on colony forming units (CFU) after incubation at 30 °C for 2 days. The remaining soil suspensions were centrifuged and filtered (0.22 μm) to spot phage dilutions on soft agar overlays of stock *R. solanacearum* QL-Rs1115 strain. After 24 h of growth at 30 °C, phage densities were calculated by counting plaques forming units (PFU).

### Culture-based validation of the effects of ‘inhibitor bacteria’ and ‘inhibitor-associated phages’ on *R. solanacearum *densities and bacterial wilt disease incidence in planta

#### Isolation of ‘inhibitor’ bacteria and ‘inhibitor-associated phages’

To validate a subset of primary and secondary phage-bacteria-pathogen interactions identified in metagenomic-based correlation analysis, we isolated non-pathogenic bacterial strains and their phages from the same field where the original rhizobox study [33] was conducted (Qilin, Nanjing, China) in July 2019 before the autumn crop season (4 years after the original rhizobox study). Nonselective agar media (NA, tryptone 5 g l^−1^, glucose 10 g l^−1^, yeast extract 0.5 g l^−1^, beef extract 3 g l^−1^, agar 25 g l^−1^, pH 7.0) were used to isolate a random selection of culturable rhizobacteria, and serial diluted soil suspensions were spread on agar plates and incubated at 30 °C for 24 h. A total of 40 bacterial colonies (candidate non-pathogen strains) were randomly selected and purified by re-streaking on new agar plates. A standard phage enrichment assay was used to isolate phages for all candidate bacteria as follows. First, all 40 candidate non-pathogen bacteria, were inoculated into NB (liquid NA) medium as monocultures and grown for 24 h with shaking at 170 rpm at 30 °C. Aliquots of bacterial cultures were then mixed independently with filtered soil suspensions (0.22 μm) containing potential phages and grown in NA media for an additional 96 h. Enriched phage-bacteria suspensions were centrifuged and filtered (0.22 μm) to remove bacteria, after soft agar overlays of individual host bacteria (1:10 mix of bacterial culture and 1% soft NA agar at 45 °C poured on top of 15% NB agar plate) were prepared and 20 μL of enriched phage suspensions spotted on top of each overlay to identify plaques of potential host-specific phages after 24 h incubation. In the end, three phages were selected due to high lytic activity and clear plaque formation on three bacterial strains that were also tested to inhibit *R. solanacearum* (see the next paragraph) and purified by streaking three times for further assays (Fig. 4 a–c). Furthermore, we selected clear plaques and examined them under a transmission electron microscope for phage (80 kV, HC-1 Hitachi TEM system, Additional file 2: Fig. S6d-f).

#### Taxonomic identification of ‘inhibitor bacteria’

The three ‘inhibitor bacteria’ were identified by Sanger sequencing the whole 16S rRNA gene using the following primer pair: 27F (5′-AGAGTTTGATCCTGGCTCAG-3′) and 1,492R (5′-GGTTACCTTGTTACGACTT-3′). Bacterial sequences were blasted against 16S ribosomal RNA sequences (bacteria and archaea) in NCBI (https://blast.ncbi.nlm.nih.gov/) to identify closely related type strains. The 16S sequences of isolated bacteria and type strains were aligned using MUSCLE [76] (UPGMB method) algorithm and relatedness visualised in the phylogenetic tree in MEGA-X (version: 10.2.2 build 10201106) [77] using the neighbour-joining method with bootstrapping (999 replicates). To determine their potential pathogenicity against tomato plants, we conducted a 7-week-long greenhouse experiment using a tomato host. We first grew a commercially available ‘Red Dwarf’ tomato cultivar (*Lycopersicon esculentum*, Shouguang-xuran Agricultural Technology Co., Ltd.) in 6-well trays filled with growth substrate (80 g substrate per well; Jiangsu-Xingnong Substrate Technology Co., Ltd., sterilised with gamma radiation before experimentation) for a week until tomatoes had reached the three-leaf stage. Seven days after planting the tomatoes, each bacterium was inoculated to the rhizosphere of 12 plant replicates using the soil drenching method at a final concentration of 10^6^~10^7^ CFU g^−1^ of substrate. All plants were grown in a greenhouse facility with a natural temperature variation ranging between 25 and 35 °C. None of the inoculated plants showed any disease symptoms at the end of the experiment (Additional file 2: Fig. S8f-h), and these ‘inhibitor bacteria’ strains were hence deemed as non-pathogenic.

#### Determining interactions between ‘inhibitor bacteria’, ‘inhibitor-associated phages’ and R. solanacearum pathogen in vitro

We used *Ralstonia solanacearum* QL-Rs1115 type strain tagged with the pYC12-mCherry plasmid [41] to measure how ‘inhibitor bacteria’ and their phages interacted with the pathogen in pairwise co-cultures in standard lab media (NB). To this end, we used a factorial design where we measured changes in the pathogen density when cultured in the absence and presence of each ‘inhibitor bacterium’, their phages or both (three-species co-cultures) with eight biological replicates each. Before the assays, all bacterial and phage species were adjusted to 10^6^~10^7^ CFU and PFU, respectively. Growth effects were quantified in 96-well microplates in 176 μL of liquid NB media at 30 °C with shaking (170 rpm). All wells were inoculated with 2 μL of *R. solanacearum*. In addition, subsets of replicates were also inoculated with 2 μL of each of the non-pathogenic bacterium both in the absence (20 μL of sterilised water; control) and presence of their phage (20 μL of phage; M.O.I = 10 where phages showed very high bacterial biomass reduction; Additional file 2: Fig. S10). The effects of non-pathogenic bacteria and their phages on *R. solanacearum* were measured using red fluorescence signal intensity (mCherry, excitation, 587 nm; emission, 610 nm) after 24 h of co-culturing using SpectraMax M5 Plate reader (Molecular Devices, Sunnyvale, CA).

#### Quantifying the effect of ‘inhibitor bacteria’ and their phages on R. solanacearum densities and bacterial wilt disease incidence in a greenhouse experiment

To determine if ‘inhibitor bacteria’ and their phages affected the *R. solanacearum* growth or bacterial wilt disease incidence in planta, we carried a separate greenhouse experiment using the same treatments as in our in vitro lab experiments using the same tomato cultivar as when determining the pathogenicity of ‘inhibitor bacteria’. Six plants per tray were considered as one biological replicate, and five replicate trays were used for each treatment, where plants were inoculated with the pathogen only, with the pathogen and each of the ‘inhibitor bacteria’, or with the pathogen and each of the ‘inhibitor bacteria’ strain and their phages, and sterile water as blank control. The pathogen was inoculated into all treatments seven days after planting tomatoes at a final concentration of 10^6^~10^7^ CFU g^−1^ of substrate. After 2 days of pathogen inoculation, non-pathogenic bacteria as well as their phages were introduced to given treatments at a final concentration of 10^6^~10^7^ CFU and 10^7^~10^8^ PFU g^−1^ (M.O.I = 10) of substrate, respectively. The position of trays was randomly rearranged twice a week and plants watered regularly using sterile water at a temperature that followed natural ambient temperature variation (25–35 °C). The experiment was finished 6 weeks after the inoculation of the pathogen, after the stabilisation of disease progression. The disease incidence was calculated as the percentage of wilted plants per replicate tray. At the end of the experiment, six plants (including three healthy and diseased per treatment) were randomly chosen and their rhizosphere soils sampled, and pathogen densities determined using qPCR as described previously [22].

### Statistical analysis

We used the one-way-ANOVA test to compare the abundance differences between treatments in both metagenomic and culture-dependent experiments. Repeated measures ANOVA was used with time-dependent data. Normal distribution and homogeneity test were tested using the Shapiro-Wilk and Bartlett tests, and log-transformed data was used when required to meet the assumptions of ANOVA [78]. In case of non-parametric data, the Wilcoxon test was used. Differences in community composition were compared using the PERMANOVA test, while the Shannon index and Bray-Curtis distances were calculated using the ‘diversity’ and ‘vegdist’ function in the vegan package based on the absolute taxa abundance matrix. The significance of Bray-Curtis distances between treatments was tested using Tukey’s multiple comparisons. For the co-occurrence analysis, data was first resampled for 1000 bootstraps before constructing computed correlation covariances based on the SparCC method [69] (two-sided pseudo-*P*-values were used to determine significant correlations). Piecewise structural equation model was conducted using the ‘psem’ function in the piecewiseSEM package. We also used the ‘calc.relimp’ function in the relaimpo package to calculate the relative importance of the three ‘functional groups’ for *R. solanacearum* density by multiple regression of linear model. SparCC analysis was conducted with python (v3.82), while all other statistical analyses were conducted using R [79] (v3.5.3) with packages and functions described in Additional file 2: Table S12. All metagenomics reads are publicly available in the SRA database under the accession number PRJNA492172 (Additional file 2: Supplementary Data 12). All code used in the analyses can be found at https://github.com/ykm7788/Microbiome2022.

## Supplementary Information

**Additional file 1:.** Supplementary data

**Additional file 2: **Table S1. Taxonomic classification of identified *Ralstonia solanacearum* phages. Table S2. Comparison of community network properties between healthy and diseased plant microbiomes. Table S3. Comparison of significance between different SEM model fits for healthy and diseased plant rhizosphere microbiome samples. Table S4. Detailed effects of different variables included in the first-step structural equation model for all five ‘functional groups’ based on Shannon alpha diversity index in healthy and diseased plant microbiome samples. Table S5. Detailed effects of different variables included in the second-step structural equation model based on Shannon alpha diversity index in healthy and diseased plant microbiome samples. Table S6. Linear model comparing the effect of ‘inhibitor-associated phage’ on ‘inhibitor bacteria’ in diseased plant microbiome samples. Formular: Inhibitor bacteria ~ Inhibitor-associated phage. Model R^2^ = 0.2909, F_1,18_ = 8.795, *P* = 0.00828. Table S7. The explanatory power of linear models for predicting *Ralstonia solanacearum* densities on each ‘functional group’ in both healthy and diseased microbiome samples. Table S8. Comparison of ‘inhibitory’ bacterial biomass in the presence and absence of their phages based on one-way ANOVA. Table S9. Comparison of *Ralstonia solanacearum* biomass between different treatments with each pair of isolated ‘inhibitor bacteria’ and ‘inhibitor-associated phage’ based on one-way ANOVA. Table S10. Comparison of tomato plant disease incidence between different treatments with each pair of isolated ‘inhibitor bacteria’ and ‘inhibitor-associated phage’ at the end of greenhouse experiment based on one-way ANOVA. Table S11. Comparison of *R. solanacearum* densities between different treatments with each pair of isolated ‘inhibitor bacteria’ and ‘inhibitor-associated phage’ at the end of greenhouse experiment based on one-way ANOVA. Table S12. Functions and packages used in R platform for statistical and bioinformatic analysis. Fig. S1. Comparison of bacterial community diversity and composition between healthy and diseased plant rhizosphere microbiome samples. a: Comparison of bacterial community diversity (Shannon index) between healthy (blue) and diseased (red) plant rhizosphere microbiome samples. Significances are shown as *: *P* < 0.05, **: *P* < 0.01, and ***: *P* < 0.001 and n.s.: no significance, one-way ANOVA for each time point (see Supplementary Data S3 for details). b: Comparison of relative bacterial abundances at the phylum level between healthy (H) and diseased (D) plant rhizosphere microbiome samples. c-g: Comparison of bacterial community composition between healthy (blue circles) and diseased (red triangles) plant rhizosphere microbiome samples at different sampling time points at bacterial species level (PCA; pairwise comparisons based on PERMANOVA). h: Bray–Curtis distances of bacterial community in healthy and diseased plant rhizosphere microbiome samples at each time point (F_4,75_ = 76.5, *P* < 0.001, Tukey’s multiple comparison after one-way ANOVA test). In panel a-g, *n* = 4 for all treatments per time point, while *n* = 16 in panel h. Fig. S2. Hierarchical composition of overall phage community based on predicted host bacterial lineages (all samples included) and distance between healthy and diseased plant microbiome. a. Circles indicate host bacterial taxonomic classifications from phylum (inner) to genus (outermost) level and percentage values show relative abundance of phages predicted to infect these bacterial taxa. b. Bray–Curtis distances of viral community in healthy and diseased plant rhizosphere microbiome samples at each time point (F_4,75_ = 22.59, *P* < 0.001, Tukey’s multiple comparison after one-way ANOVA test) and the correlation with time (week 3-6, linear model). Fig. S3. Comparison of initial soil physicochemical properties between healthy and diseased plant samples. Significances are shown as *: *P* < 0.05 and n.s.: no significance, one-way ANOVA for each pair (*n* = 4, see Supplementary Data S3 for details). Fig. S4. Comparison of *R. solanacearum-*specific phage abundances between healthy and diseased plant rhizosphere microbiome sample replicates at different time points. Each pair of panels (a-h) show phage density dynamics for diseased and healthy plant rhizosphere microbiome samples and different line colours show individual plant replicates. Line symbols denote for different viral families as denoted in the legend. Phages RSF1, RSL2 and P4282 had relatively higher abundances in early samples, while phage P4282 persistently decreased in abundance, phages RSF1 and RSL2 edged up towards the end of the experiment in both diseased and healthy plant microbiome samples, respectively. In contrast, phages RSB3 and RS138 showed steady increase in their relative abundances in part of healthy plant microbiome samples. In addition, phages RSL1, RSK1 and RSJ5 showed multiple peak stages in different plant rhizosphere in both healthy and diseased samples. Fig. S5. Comparisons of interactions between ‘functional groups’ and pathogen densities in healthy and diseased plant rhizosphere microbiomes. a-b: Difference in the proportion of significant correlations between ‘functional groups’ and pathogen densities in healthy (a) and diseased (b) plant rhizosphere microbiomes. Light and dark blue colours denote for ‘inhibitor bacteria’ and ‘inhibitor-associated phages’, while light and dark green colours denote for ‘facilitator bacteria’ and ‘facilitator-associated phages’. c-e: Structural equation models based on different community indexes (Shannon index, Simpson index and average abundance) illustrating primary-phage-effect (*R. solanacearum*-specific phage to *R. solanacearum*), primary-bacteria effects (‘inhibitor bacteria’ and ‘facilitator bacteria’ to *R. solanacearum*) as well as secondary-phage effects (‘inhibitor-associated phages’ and ‘facilitator-associated phages’ to ‘inhibitor bacteria’ and ‘facilitator bacteria’, respectively and to *R. solanacearum*) in both healthy and diseased plant microbiomes. Fig. S6. Comparisons of secondary metabolism synthesis related gene abundances between healthy (blue) and diseased (red) sample pairs in week 6. Statistical significance between treatments was determined by one-way ANOVA test with *: *P* < 0.05, **: *P* < 0.01, *n* = 4 for all treatments (see Supplementary Data S3 for details). Fig. S7. Taxonomic classification of isolated ‘inhibitor bacteria’ and transmission electron microscope photograph of their isolated phages. a-c: Phylogenetic tree of three inhibitor bacterial strains based on 16s rRNA sequences using neighbour-joining method (on bold). The evolutionary distances were computed using the Maximum Composite Likelihood method and the scale bar indicates the average number of amino acid substitutions per site. d-f: TEM of representative isolates performed by HC-1 Hitachi TEM system at 80 kV. Fig. S8. Host bacterium abundance examined with (light blue) or without (dark blue) associated phage after co-culturing 24 h. Statistical significance between treatments was determined by one-way ANOVA test with ***:*P* < 0.001. Error bar: standard deviation. *n* = 8 for all treatments (see Supplementary Table S8 for details). Fig. S9. Photograph of tomato plants at the end of greenhouse experiment. a-c: Treatments with plants inoculated with *R. solanacearum* and different ‘inhibitor bacteria’ in the presence (right) and absence (left) of ‘inhibitor-associated phages’. d: ‘*R. solanacearum*-only’ control plants inoculated only with *R. solanacearum*. e: Blank control inoculated only with sterile water without any bacteria or phages. f-h: Treatments with plants inoculated with different ‘inhibitor bacteria’ only. In panels a-d, DI represents average disease incidence and diseased plants are highlighted with red flags. No disease symptoms were observed in e-h. Fig. S10. Comparison of the relative bacterial biomass reduction at different initial multiplicity of infections (M.O.I.). With all‘inhibitor-associated phages’, highest bacterial biomass reduction was observed with M.O.I = 10. Statistical significance between treatments was determined by one-way ANOVA test with *: *P* < 0.05, **: *P* < 0.01, ***: *P* < 0.001, *n* = 8 for all treatments

## Data Availability

All data has been made publicly available as stated in the manuscript.
