# Supplementary material for: Rhizosphere phage communities drive soil suppressiveness to bacterial wilt disease
Source: Microbiome. 2023 Feb 1;11:16. doi: 10.1186/s40168-023-01463-8 (PMC9890766; doi:10.1186/s40168-023-01463-8)
Supplement: Supplementary file 3 — Additional file 2: Table S1. Taxonomic classification of identified Ralstonia solanacearum phages. Table S2. Comparison of community network properties between healthy and diseased plant microbiomes. Table S3. Comparison of significance between different SEM model fits for healthy and diseased plant rhizosphere microbiome samples. Table S4. Detailed effects of different variables included in the first-step structural equation model for all five ‘functional groups’ based on Shannon alpha diversity index in healthy and diseased plant microbiome samples. Table S5. Detailed effects of different variables included in the second-step structural equation model based on Shannon alpha diversity index in healthy and diseased plant microbiome samples. Table S6. Linear model comparing the effect of ‘inhibitor-associated phage’ on ‘inhibitor bacteria’ in diseased plant microbiome samples. Formular: Inhibitor bacteria ~ Inhibitor-associated phage. Model R2 = 0.2909, F1,18 = 8.795, P = 0.00828. Table S7. The explanatory power of linear models for predicting Ralstonia solanacearum densities on each ‘functional group’ in both healthy and diseased microbiome samples. Table S8. Comparison of ‘inhibitory’ bacterial biomass in the presence and absence of their phages based on one-way ANOVA. Table S9. Comparison of Ralstonia solanacearum biomass between different treatments with each pair of isolated ‘inhibitor bacteria’ and ‘inhibitor-associated phage’ based on one-way ANOVA. Table S10. Comparison of tomato plant disease incidence between different treatments with each pair of isolated ‘inhibitor bacteria’ and ‘inhibitor-associated phage’ at the end of greenhouse experiment based on one-way ANOVA. Table S11. Comparison of R. solanacearum densities between different treatments with each pair of isolated ‘inhibitor bacteria’ and ‘inhibitor-associated phage’ at the end of greenhouse experiment based on one-way ANOVA. Table S12. Functions and packages used in R platform for statistical and [file 40168_2023_1463_MOESM2_ESM.docx]

Supplementary information for:

**Rhizosphere phage communities drive soil suppressiveness to bacterial wilt disease**

Keming Yang^1,2^, Xiaofang Wang^1^, Rujiao Hou^1^, Jingxuan Li^1^, Chunxia Lu^1^, Shuo Wang^1^, Yangchun Xu^1^, Qirong Shen^1^, Ville-Petri Friman^1,2*^and Zhong Wei^1*^

^1^Laboratory of Bio-interactions and Crop Health, Jiangsu Provincial Key Lab of Solid Organic Waste Utilization, Jiangsu Collaborative Innovation Center of Solid Organic Wastes, Educational Ministry Engineering Center of Resource-saving fertilizers, Nanjing Agricultural University, Nanjing 210095, Jiangsu, P.R. China

^2^University of York, Department of Biology, Wentworth Way, York, YO10 5DD, UK

*Correspondence: weizhong@njau.edu.cn (Zhong Wei); ville.friman@york.ac.uk (Ville-Petri Friman)

**Supplementary Tables**

**Supplementary Table 1.** Taxonomic classification of identified *Ralstonia solanacearum* phages

| Phage name | Family | Accession ID |
| --- | --- | --- |
| P4282 | unclassified | AB048798 |
| RS138 | Siphoviridae | NC_029107.1 |
| RSB3 | Podoviridae | NC_022917.1 |
| RSF1 | Myoviridae | NC_028899.1 |
| RSJ5 | Podoviridae | NC_029007.1 |
| RSK1 | Podoviridae | NC_022915.1 |
| RSL1 | Myoviridae | NC_010811.2 |
| RSL2 | Myoviridae | NC_028950.1 |

**Supplementary Table 2.** Comparison of community network properties between healthy and diseased plant microbiomes.

| Property | Healthy | Diseased |
| --- | --- | --- |
| nodes | 139 | 137 |
| edges | 140 | 138 |
| connectance | 0.0142 | 0.0145 |
| connectivity | 1 | 1 |
| betweenness centralization | 0.9685 | 0.9675 |
| Degree centralization | 0.3454 | 0.3432 |

**Supplementary Table 3.** Comparison of significance between different SEM model fits for healthy and diseased plant rhizosphere microbiome samples.

| Index | Outcome | Facilitator species | Fisher's C | *P*-value | AIC |
| --- | --- | --- | --- | --- | --- |
| Shannon diversity | Healthy | include | 13.88 | 0.179 | 39.88 |
|  | Diseased | include | 24.714 | 0.006 | 50.71 |
|  | Healthy | exclude | 1.782 | 0.41 | 17.78 |
|  | Diseased | exclude | 9.962 | 0.007 | 25.962 |
| Simpson diversity | Healthy | include | 28.2 | 0.002 | 54.2 |
|  | Diseased | include | 35.88 | < 0.001 | 61.88 |
| Average abundance | Healthy | include | 35.27 | < 0.001 | 61.27 |
|  | Diseased | include | 57.82 | < 0.001 | 83.82 |

**Supplementary Table 4.** Detailed effects of different variables included in the first-step structural equation model for all five ‘functional groups’ based on Shannon alpha diversity index in healthy and diseased plant microbiome samples.

| Model | Response | Predictor | Estimate | Std.Error | DF | Crit.Value | *P*-value | Std.Estimate |
| --- | --- | --- | --- | --- | --- | --- | --- | --- |
| Healthy | Facilitator bacteria | Facilitator-associated phage | 0.1073 | 0.0549 | 18 | 1.9548 | 0.0663 | 0.4185 |
| Healthy | Inhibitor bacteria | Inhibitor-associated phage | 0.0366 | 0.039 | 18 | 0.9392 | 0.3601 | 0.2161 |
| Healthy | Pathogen | Pathogen phage | -1.4561 | 1.3343 | 14 | -1.0913 | 0.2936 | -0.2736 |
| Healthy | Pathogen | Facilitator bacteria | -3.1557 | 6.0119 | 14 | -0.5249 | 0.6079 | -0.1262 |
| Healthy | Pathogen | Facilitator-associated phage | -1.9489 | 1.5672 | 14 | -1.2436 | 0.2341 | -0.3039 |
| Healthy | Pathogen | Inhibitor bacteria | -27.0968 | 10.7241 | 14 | -2.5267 | 0.0242 | -0.4813 |
| Healthy | Pathogen | Inhibitor-associated phage | 1.3656 | 2.3726 | 14 | 0.5756 | 0.574 | 0.1432 |
| Diseased | Facilitator bacteria | Facilitator-associated phage | 0.1077 | 0.0268 | 18 | 4.0194 | 0.0008 | 0.6877 |
| Diseased | Inhibitor bacteria | Inhibitor-associated phage | -0.252 | 0.085 | 18 | -2.9657 | 0.0083 | -0.5729 |
| Diseased | Pathogen | Pathogen phage | 2.3579 | 2.5277 | 14 | 0.9328 | 0.3667 | 0.3715 |
| Diseased | Pathogen | Facilitator bacteria | -3.9476 | 12.5155 | 14 | -0.3154 | 0.7571 | -0.106 |
| Diseased | Pathogen | Facilitator-associated phage | -4.8213 | 3.1227 | 14 | -1.5439 | 0.1449 | -0.8272 |
| Diseased | Pathogen | Inhibitor bacteria | 0.1777 | 6.9383 | 14 | 0.0256 | 0.9799 | 0.0093 |
| Diseased | Pathogen | Inhibitor-associated phage | 0.9376 | 3.195 | 14 | 0.2935 | 0.7735 | 0.1122 |

**Supplementary Table 5.** Detailed effects of different variables included in the second-step structural equation model based on Shannon alpha diversity index in healthy and diseased plant microbiome samples.

| Model | Response | Predictor | Estimate | Std.Error | DF | Crit.Value | *P*-value | Std.Estimate |
| --- | --- | --- | --- | --- | --- | --- | --- | --- |
| Healthy | Inhibitor bacteria | Inhibitor-associated phage | 0.0366 | 0.039 | 18 | 0.9392 | 0.3601 | 0.2161 |
| Healthy | Pathogen | Pathogen phage | -2.3161 | 1.0773 | 16 | -2.1499 | 0.0472 | -0.4352 |
| Healthy | Pathogen | Inhibitor bacteria | -24.0043 | 10.374 | 16 | -2.3139 | 0.0343 | -0.4263 |
| Healthy | Pathogen | Inhibitor-associated phage | -0.461 | 1.9012 | 16 | -0.2425 | 0.8115 | -0.0484 |
| Diseased | Inhibitor bacteria | Inhibitor-associated phage | -0.252 | 0.085 | 18 | -2.9657 | 0.0083 | -0.5729 |
| Diseased | Pathogen | Pathogen phage | 0.327 | 2.1038 | 16 | 0.1555 | 0.8784 | 0.0515 |
| Diseased | Pathogen | Inhibitor bacteria | 3.3206 | 6.4777 | 16 | 0.5126 | 0.6152 | 0.1748 |
| Diseased | Pathogen | Inhibitor-associated phage | -2.9638 | 2.3417 | 16 | -1.2656 | 0.2238 | -0.3546 |

**Supplementary Table 6.** Linear model comparing the effect of ‘inhibitor-associated phage’ on ‘inhibitor bacteria’ in diseased plant microbiome samples. Formular: Inhibitor bacteria ~ Inhibitor-associated phage. Model R^2^ = 0.2909, F_1,18_ = 8.795, *P* = 0.00828.

|  | Estimate | Std. Error | t value | Pr(>\|t\|) |
| --- | --- | --- | --- | --- |
| (Intercept) | 2.72535 | 0.25543 | 10.67 | 3.26E-09 |
| Inhibitor-associated phage | -0.25205 | 0.08499 | -2.966 | 0.00828 |

**Supplementary Table 7.** The explanatory power of linear models for predicting *Ralstonia solanacearum* densities on each ‘functional group’ in both healthy and diseased microbiome samples.

| Sample type | Functional group | Power |
| --- | --- | --- |
| Healthy microbiome | *R. solanacearum*-phage | 0.226536 |
|  | Inhibitor bacteria | 0.227664 |
|  | Inhibitor-associated phage | 0.048837 |
| Diseased microbiome | *R. solanacearum-*phage | 0.024285 |
|  | Inhibitor bacteria | 0.053139 |
|  | Inhibitor-associated phage | 0.119828 |

**Supplementary Table 8.** Comparison of ‘inhibitory’ bacterial biomass in the presence and absence of their phages based on one-way ANOVA.

| Strain | Treatments | | Df | SumSq | MeanSq | F-value | Pr(>F) |
| --- | --- | --- | --- | --- | --- | --- | --- |
| YL-Ste-01 | without phage | with phage | 1 | 1.243 | 1.2427 | 135.9 | 1.35E-08 |
|  |  |  | 14 | 0.128 | 0.0091 |  |  |
| YL-Bac-29 |  |  | 1 | 0.586 | 0.586 | 77.21 | 4.52E-07 |
|  |  |  | 14 | 0.1062 | 0.0076 |  |  |
| YL-Ent-31 |  |  | 1 | 0.3347 | 0.3347 | 40.88 | 1.67E-05 |
|  |  |  | 14 | 0.1146 | 0.0082 |  |  |

**Supplementary Table 9.** Comparison of *Ralstonia solanacearum* biomass between different treatments with each pair of isolated ‘inhibitor bacteria’ and ‘inhibitor-associated phage’ based on one-way ANOVA.

| Inhibitor strain | Treatments | | Df | SumSq | MeanSq | F-value | Pr(>F) |
| --- | --- | --- | --- | --- | --- | --- | --- |
| YL-Ste-01 | Inhibitor bacteria | Inhibitor bacteria  +Inhibitor-associated phage | 1 | 49356 | 49356 | 145.4 | 8.78E-09 |
|  |  |  | 14 | 4752 | 339 |  |  |
|  | *R. solanacearum*  -mono | Inhibitor bacteria  +Inhibitor-associated phage | 1 | 602186 | 602186 | 700.8 | 2.34E-13 |
|  |  |  | 14 | 12029 | 859 |  |  |
|  | *R. solanacearum*  -mono | Inhibitor-associated phage | 1 | 32 | 32 | 0.025 | 0.877 |
|  |  |  | 14 | 18105 | 1293 |  |  |
|  | *R. solanacearum*  -mono | Inhibitor bacteria | 1 | 996340 | 996340 | 1652 | 6.21E-16 |
|  |  |  | 14 | 8441 | 603 |  |  |
| YL-Bac-29 | Inhibitor bacteria | Inhibitor bacteria  +Inhibitor-associated phage | 1 | 22022 | 22022 | 17.8 | 0.000858 |
|  |  |  | 14 | 17322 | 1237 |  |  |
|  | *R. solanacearum*  -mono | Inhibitor bacteria  +Inhibitor-associated phage | 1 | 421833 | 421833 | 258.3 | 2.04E-10 |
|  |  |  | 14 | 22865 | 1633 |  |  |
|  | *R. solanacearum*  -mono | Inhibitor-associated phage | 1 | 1018 | 1018 | 1.356 | 0.264 |
|  |  |  | 14 | 10510 | 750.7 |  |  |
|  | *R. solanacearum*  -mono | Inhibitor bacteria | 1 | 636621 | 636621 | 875.9 | 5.03E-14 |
|  |  |  | 14 | 10175 | 727 |  |  |
| YL-Ent-31 | Inhibitor bacteria | Inhibitor bacteria  +Inhibitor-associated phage | 1 | 24507 | 24507 | 99.95 | 9.37E-08 |
|  |  |  | 14 | 3433 | 245 |  |  |
|  | *R. solanacearum*  -mono | Inhibitor bacteria  +Inhibitor-associated phage | 1 | 533376 | 533376 | 702.9 | 2.29E-13 |
|  |  |  | 14 | 10624 | 759 |  |  |
|  | *R. solanacearum*  -mono | Inhibitor-associated phage | 1 | 663 | 662.5 | 0.661 | 0.43 |
|  |  |  | 14 | 14038 | 1002.7 |  |  |
|  | *R. solanacearum*  -mono | Inhibitor bacteria | 1 | 786546 | 786546 | 1291 | 3.44E-15 |
|  |  |  | 14 | 8528 | 609 |  |  |

**Supplementary Table 10.** Comparison of tomato plant disease incidence between different treatments with each pair of isolated ‘inhibitor bacteria’ and ‘inhibitor-associated phage’ at the end of greenhouse experiment based on one-way ANOVA.

| Inhibitor strain | Treatments | | Df | SumSq | MeanSq | F-value | Pr(>F) |
| --- | --- | --- | --- | --- | --- | --- | --- |
| YL-Ste-01 | Inhibitor bacteria | Inhibitor bacteria  +Inhibitor-associated phage | 1 | 0.625 | 0.625 | 18.75 | 0.00251 |
|  |  |  | 8 | 0.2667 | 0.0333 |  |  |
|  | *R. solanacearum*  -mono | Inhibitor bacteria | 1 | 0.4 | 0.4 | 12 | 0.00852 |
|  |  |  | 8 | 0.2667 | 0.0333 |  |  |
| YL-Bac-29 | Inhibitor bacteria | Inhibitor bacteria  +Inhibitor-associated phage | 1 | 0.4 | 0.4 | 10.29 | 0.0125 |
|  |  |  | 8 | 0.3111 | 0.0389 |  |  |
|  | *R. solanacearum*  -mono | Inhibitor bacteria | 1 | 0.4694 | 0.4694 | 16.9 | 0.00339 |
|  |  |  | 8 | 0.2222 | 0.0278 |  |  |
| YL-Ent-31 | Inhibitor bacteria | Inhibitor bacteria  +Inhibitor-associated phage | 1 | 0.04444 | 0.04444 | 1.6 | 0.242 |
|  |  |  | 8 | 0.22222 | 0.02778 |  |  |
|  | *R. solanacearum*  -mono | Inhibitor bacteria | 1 | 0.5444 | 0.5444 | 26.13 | 0.000916 |
|  |  |  | 8 | 0.1667 | 0.0208 |  |  |

**Supplementary Table 11.** Comparison of *R. solanacearum* densities between different treatments with each pair of isolated ‘inhibitor bacteria’ and ‘inhibitor-associated phage’ at the end of greenhouse experiment based on one-way ANOVA.

| Inhibitor strain | Treatments | | Df | SumSq | MeanSq | F-value | Pr(>F) |
| --- | --- | --- | --- | --- | --- | --- | --- |
| YL-Ste-01 | Inhibitor bacteria | Inhibitor bacteria +Inhibitor-associated phage | 1 | 2.782 | 2.7817 | 24.86 | 0.000549 |
|  |  |  | 10 | 1.119 | 0.1119 |  |  |
|  | *R. solanacearum*  -mono | Inhibitor bacteria +Inhibitor-associated phage | 1 | 6.442 | 6.442 | 32.77 | 0.000192 |
|  |  |  | 10 | 1.966 | 0.197 |  |  |
|  | *R. solanacearum*  -mono | Inhibitor bacteria | 1 | 17.69 | 17.69 | 72.16 | 6.93E-06 |
|  |  |  | 10 | 2.451 | 0.245 |  |  |
| YL-Bac-29 | Inhibitor bacteria | Inhibitor bacteria +Inhibitor-associated phage | 1 | 4.031 | 4.031 | 21.58 | 0.000915 |
|  |  |  | 10 | 1.868 | 0.187 |  |  |
|  | *R. solanacearum*  -mono | Inhibitor bacteria +Inhibitor-associated phage | 1 | 7.422 | 7.422 | 22.08 | 0.000843 |
|  |  |  | 10 | 3.362 | 0.336 |  |  |
|  | *R. solanacearum*  -mono | Inhibitor bacteria | 1 | 22.393 | 22.39 | 124.1 | 5.87E-07 |
|  |  |  | 10 | 1.805 | 0.18 |  |  |
| YL-Ent-31 | Inhibitor bacteria | Inhibitor bacteria +Inhibitor-associated phage | 1 | 2.182 | 2.182 | 51.42 | 3.03E-05 |
|  |  |  | 10 | 0.4243 | 0.0424 |  |  |
|  | *R. solanacearum*  -mono | Inhibitor bacteria +Inhibitor-associated phage | 1 | 9.948 | 9.948 | 51.49 | 3.01E-05 |
|  |  |  | 10 | 1.932 | 0.193 |  |  |
|  | *R. solanacearum*  -mono | Inhibitor bacteria | 1 | 21.448 | 21.448 | 119.8 | 6.92E-07 |
|  |  |  | 10 | 1.791 | 0.179 |  |  |

**Supplementary Table 12.** Functions and packages used in R platform for statistical and bioinformatic analysis.

| Analysis | Function | R package |
| --- | --- | --- |
| normality test | shapiro.test | stats |
| homogeneity test of variance | bartlett.test | stats |
| one-way ANOVA | aov | stats |
| Wilcoxon non-parametric test | wilcox.test | stats |
| Tukey’s multiple comparison | HSD.test | agricolae |
| PCA | prcomp | stats |
| PERMANOVA | adonis2 | vegan |
| linear correlation | lm | stats |
| generalized linear model | glm | stats |
| Shannon index | diversity | vegan |
| Simpson index | diversity | vegan |
| Bray–Curtis distance | vegdist | vegan |
| chao1 richness | chao1 | fossil |
| Nodes | E | igraph |
| Edges | V | igraph |
| Density | edge_density | igraph |
| Connectivity | edge_connectivity | igraph |
| Betweenness centralization | centralization.betweenness | igraph |
| Degree centralization | centralization.degree | igraph |
| piecewise SEM | psem | piecewiseSEM |
| Explanatory power | calc.relimp | relaimpo |

**Supplementary Figures**

**Supplementary Figure 1**


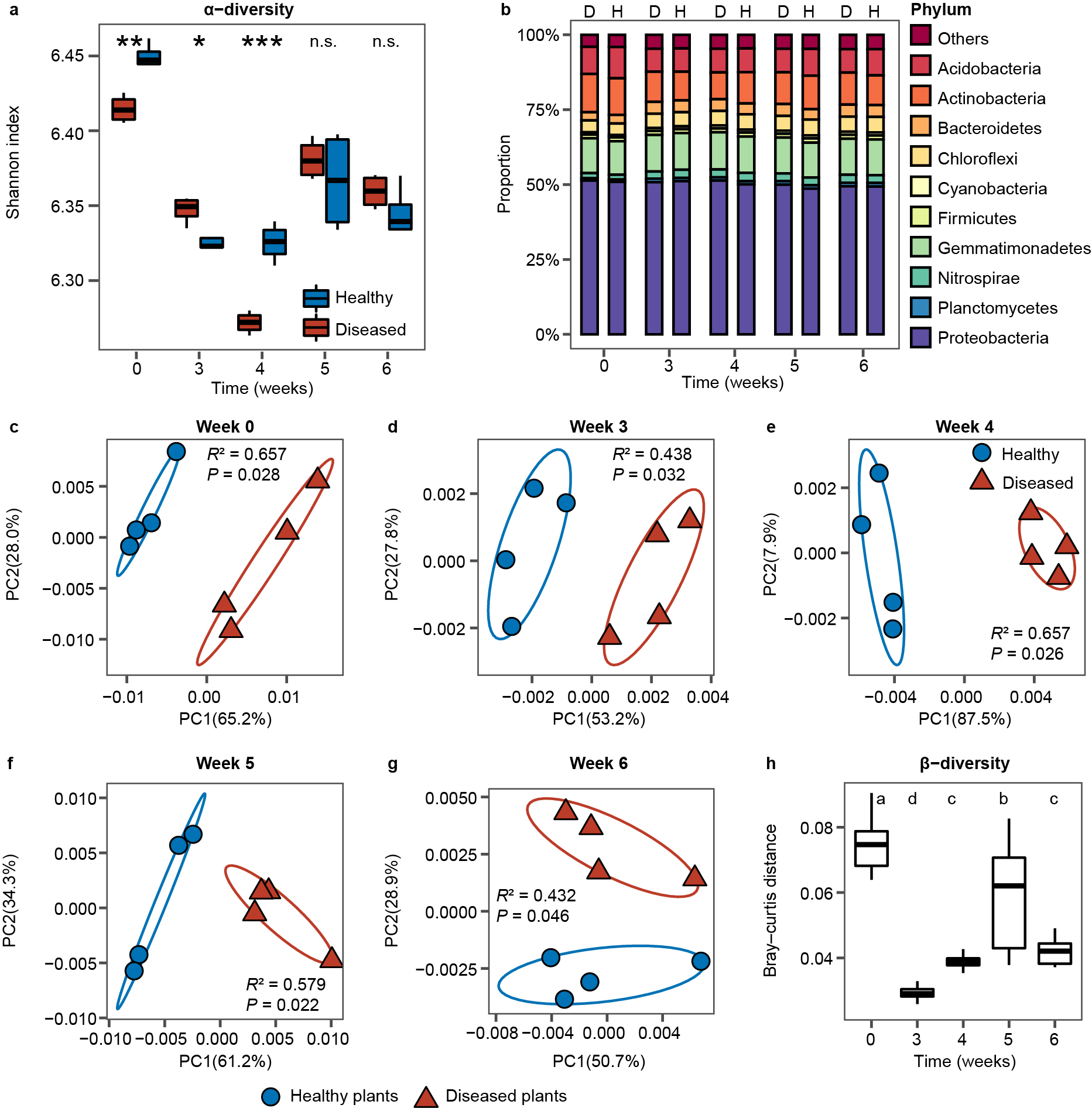


**Supplementary Figure 1. Comparison of bacterial community diversity and composition between healthy and diseased plant rhizosphere microbiome samples. a:** Comparison of bacterial community diversity (Shannon index) between healthy (blue) and diseased (red) plant rhizosphere microbiome samples. Significances are shown as *: *P* < 0.05, **: *P* < 0.01, and ***: *P* < 0.001 and n.s.: no significance, one-way ANOVA for each time point (see Supplementary Data S3 for details). **b:** Comparison of relative bacterial abundances at the phylum level between healthy (H) and diseased (D) plant rhizosphere microbiome samples. **c-g:** Comparison of bacterial community composition between healthy (blue circles) and diseased (red triangles) plant rhizosphere microbiome samples at different sampling time points at bacterial species level (PCA; pairwise comparisons based on PERMANOVA). **h:** Bray–Curtis distances of bacterial community in healthy and diseased plant rhizosphere microbiome samples at each time point (F_4,75_ = 76.5, *P* < 0.001, Tukey’s multiple comparison after one-way ANOVA test). In panel **a**-**g**, *n* = 4 for all treatments per time point, while *n* = 16 in panel **h**.

**Supplementary Figure 2**


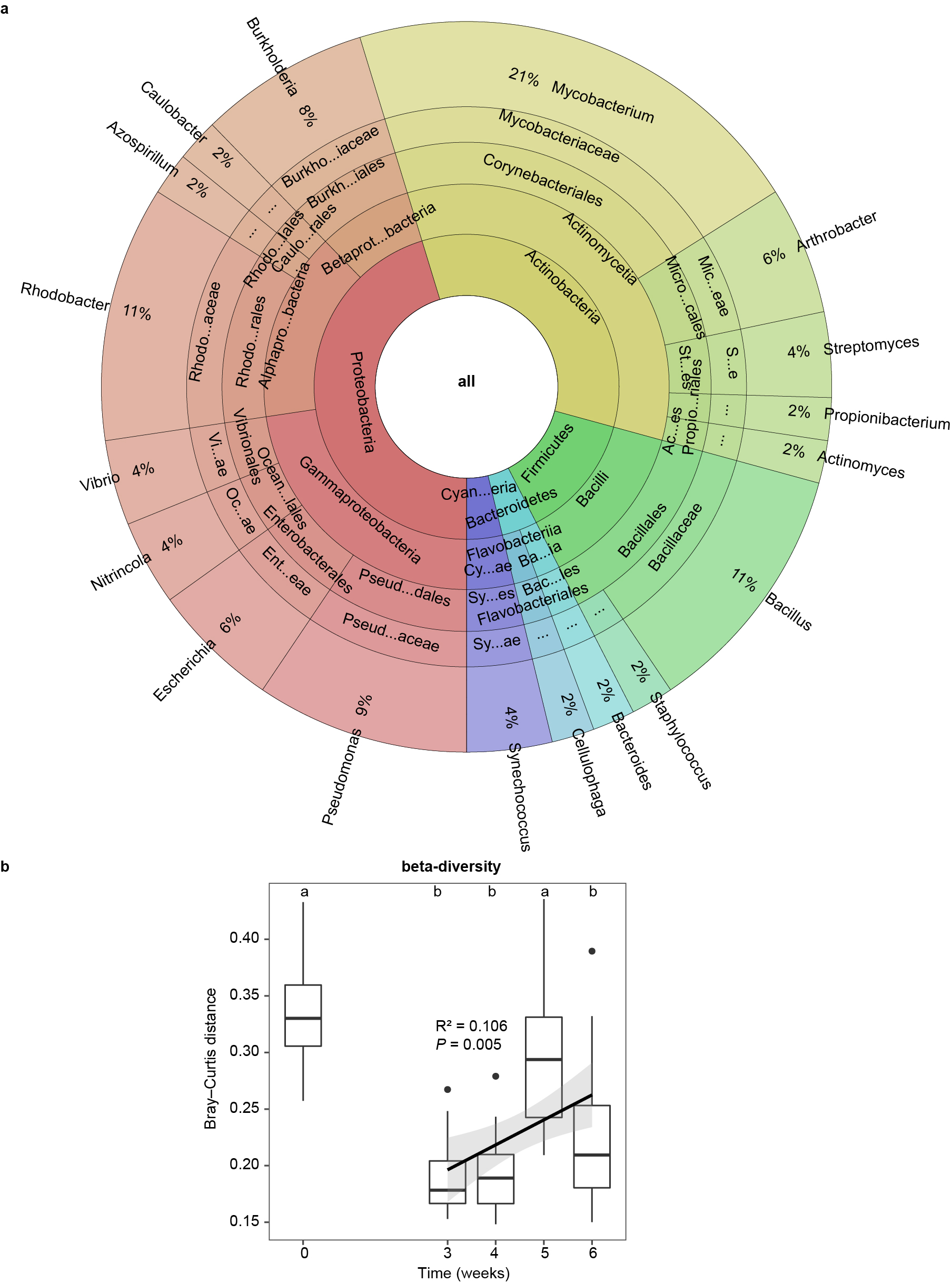


**Supplementary Figure 2. Hierarchical composition of overall phage community based on predicted host bacterial lineages (all samples included) and distance between healthy and diseased plant microbiome.** **a.** Circles indicate host bacterial taxonomic classifications from phylum (inner) to genus (outermost) level and percentage values show relative abundance of phages predicted to infect these bacterial taxa. **b.** Bray–Curtis distances of viral community in healthy and diseased plant rhizosphere microbiome samples at each time point (F_4,75_ = 22.59, *P* < 0.001, Tukey’s multiple comparison after one-way ANOVA test) and the correlation with time (week 3-6, linear model).

**Supplementary Figure 3**


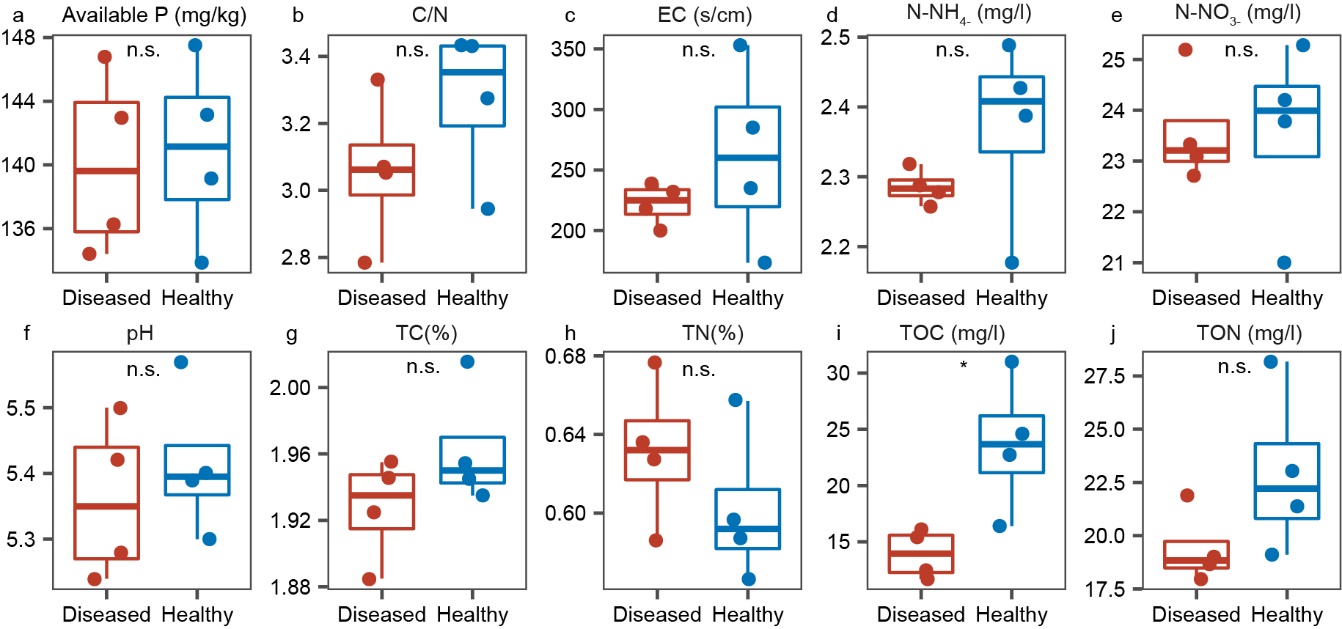


**Supplementary Figure 3. Comparison of initial soil physicochemical properties between healthy and diseased plant samples.** Significances are shown as *: *P* < 0.05 and n.s.: no significance, one-way ANOVA for each pair (*n* = 4, see Supplementary Data S3 for details).

**Supplementary Figure 4**


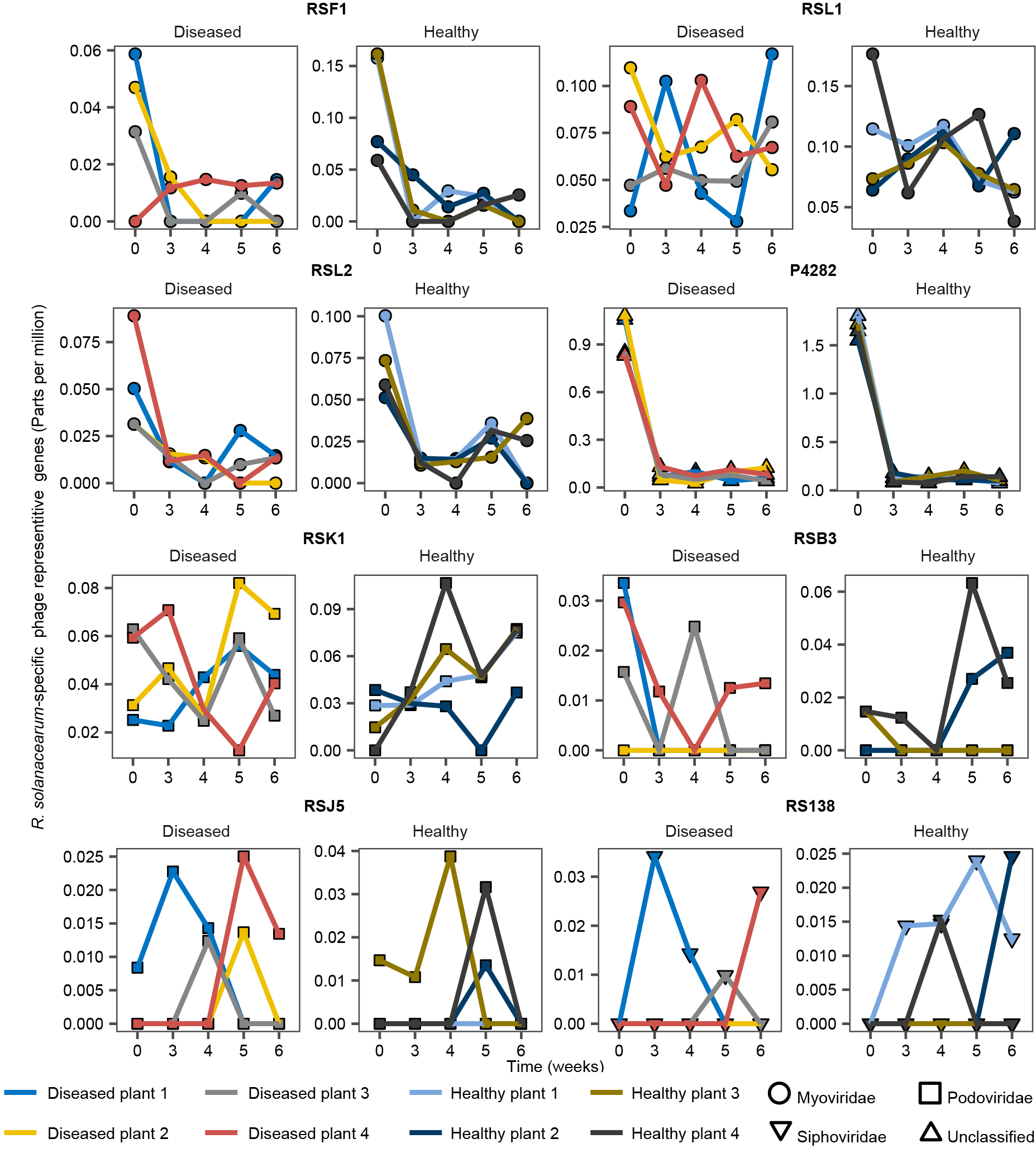


**Supplementary Figure 4. Comparison of *R. solanacearum-*specific phage abundances between healthy and diseased plant rhizosphere microbiome sample replicates at different time points.** Each pair of panels (a-h) show phage density dynamics for diseased and healthy plant rhizosphere microbiome samples and different line colors show individual plant replicates. Line symbols denote for different viral families as denoted in the legend. Phages RSF1, RSL2 and P4282 had relatively higher abundances in early samples, while phage P4282 persistently decreased in abundance, phages RSF1 and RSL2 edged up towards the end of the experiment in both diseased and healthy plant microbiome samples, respectively. In contrast, phages RSB3 and RS138 showed steady increase in their relative abundances in part of healthy plant microbiome samples. In addition, phages RSL1, RSK1 and RSJ5 showed multiple peak stages in different plant rhizosphere in both healthy and diseased samples.

**Supplementary Figure 5**


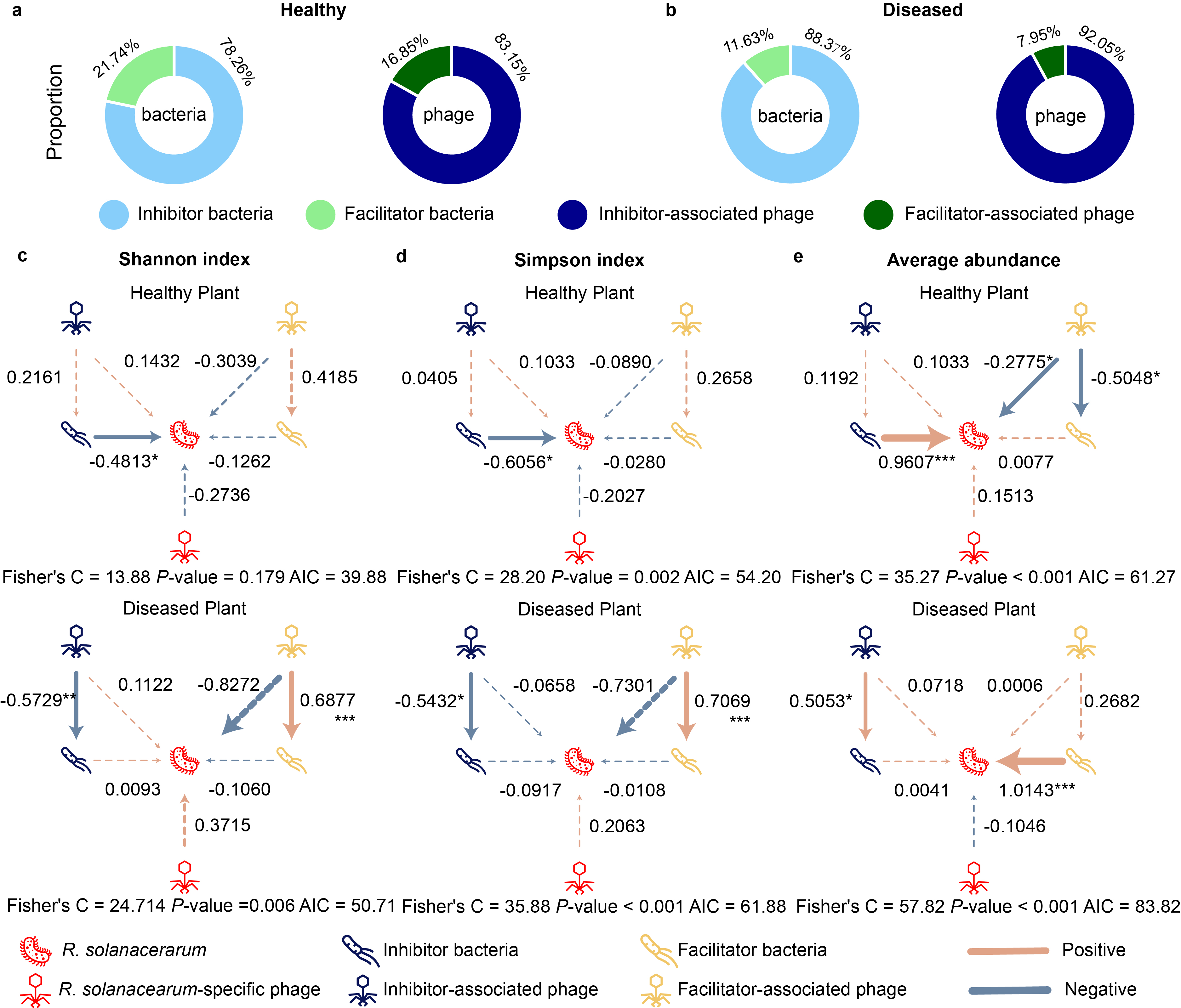


**Supplementary Figure 5. Comparisons of interactions between ‘functional groups’ and pathogen densities in healthy and diseased plant rhizosphere microbiomes. a-b**: Difference in the proportion of significant correlations between ‘functional groups’ and pathogen densities in healthy (**a**) and diseased (**b**) plant rhizosphere microbiomes. Light and dark blue colors denote for ‘inhibitor bacteria’ and ‘inhibitor-associated phages’, while light and dark green colors denote for ‘facilitator bacteria’ and ‘facilitator-associated phages’. **c-e**: Structural equation models based on different community indexes (Shannon index, Simpson index and average abundance) illustrating primary-phage-effect (*R. solanacearum*-specific phage to *R. solanacearum*), primary-bacteria effects (‘inhibitor bacteria’ and ‘facilitator bacteria’ to *R. solanacearum*) as well as secondary-phage effects (‘inhibitor-associated phages’ and ‘facilitator-associated phages’ to ‘inhibitor bacteria’ and ‘facilitator bacteria’, respectively and to *R. solanacearum*) in both healthy and diseased plant microbiomes.

**Supplementary Figure 6**

**
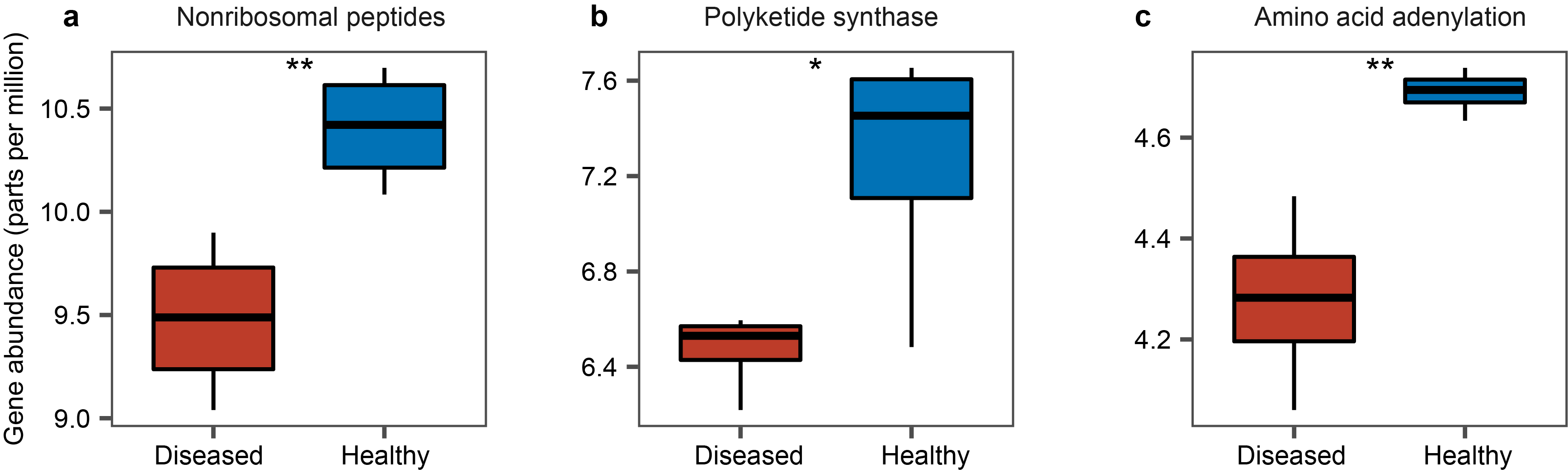
**

**Supplementary Figure 6. Comparisons of secondary metabolism synthesis related gene abundances between healthy (blue) and diseased (red) sample pairs in week 6.** Statistical significance between treatments was determined by one-way ANOVA test with *: *P* < 0.05, **: *P* < 0.01, *n* = 4 for all treatments (see Supplementary Data S3 for details).

**Supplementary Figure 7**


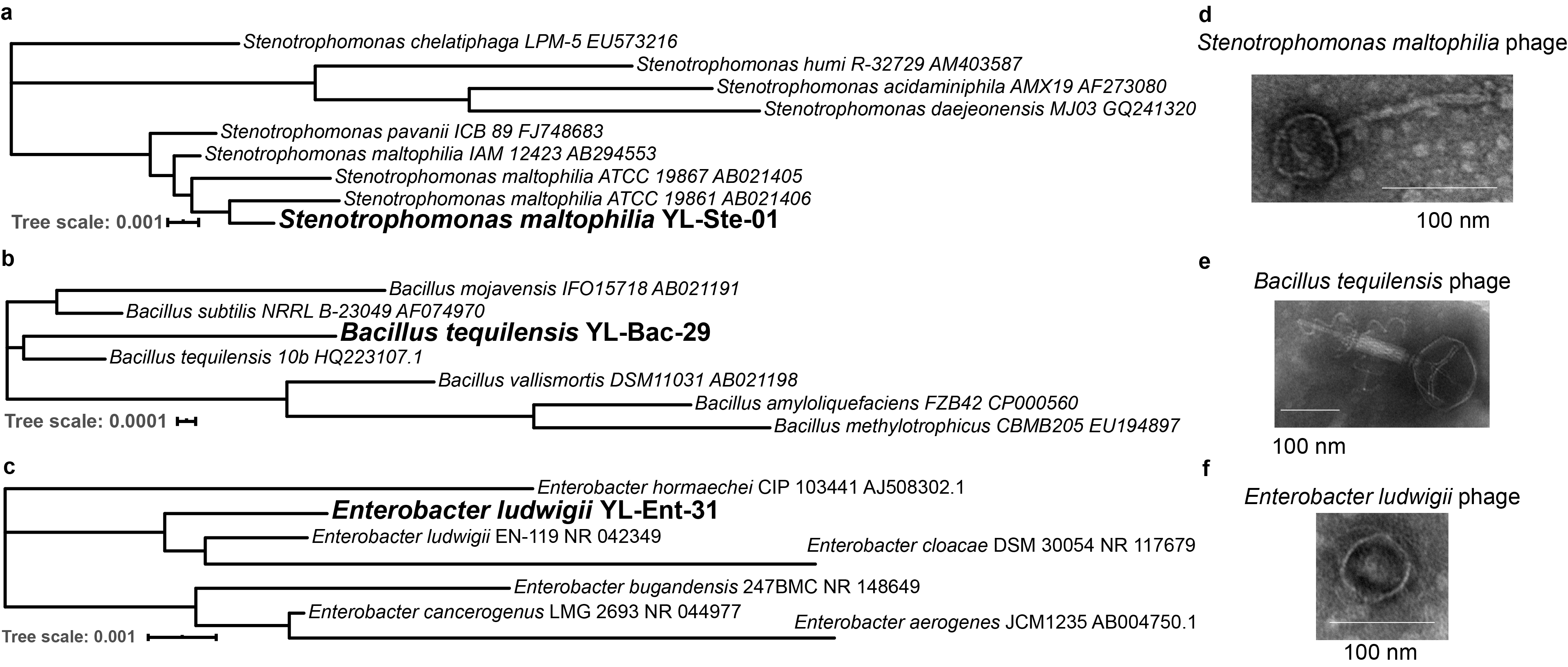


**Supplementary Figure 7. Taxonomic classification of isolated ‘inhibitor bacteria’ and transmission electron microscope photograph of their isolated phages. a-c:** Phylogenetic tree of three inhibitor bacterial strains based on 16s rRNA sequences using neighbor-joining method (on bold). The evolutionary distances were computed using the Maximum Composite Likelihood method and the scale bar indicates the average number of amino acid substitutions per site. **d-f**: TEM of representative isolates performed by HC-1 Hitachi TEM system at 80 kV.

**Supplementary Figure 8**


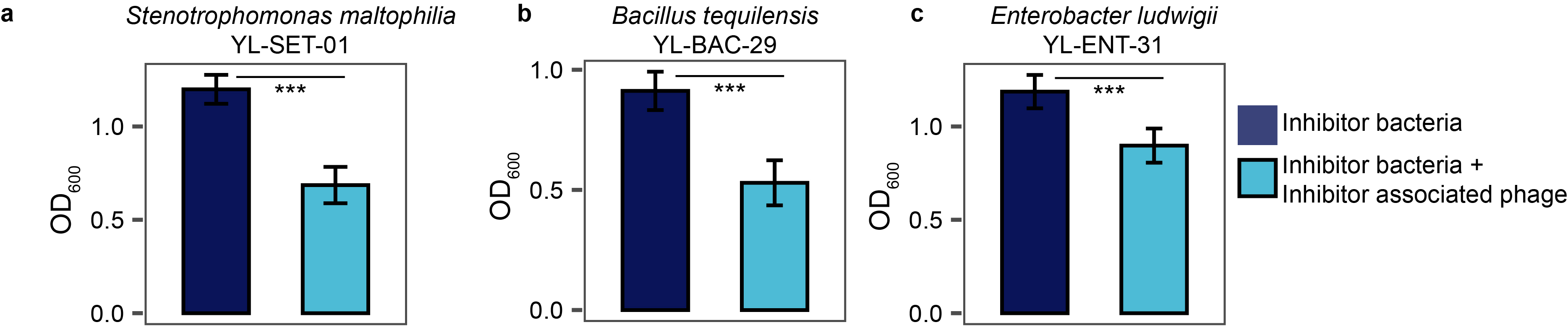


**Supplementary Figure 8. Host bacterium abundance examined with (light blue) or without (dark blue) associated phage after co-culturing 24 hours.** Statistical significance between treatments was determined by one-way ANOVA test with ***:*P* < 0.001. Error bar: standard deviation. *n* = 8 for all treatments (see Supplementary Table S8 for details).

**Supplementary Figure 9**


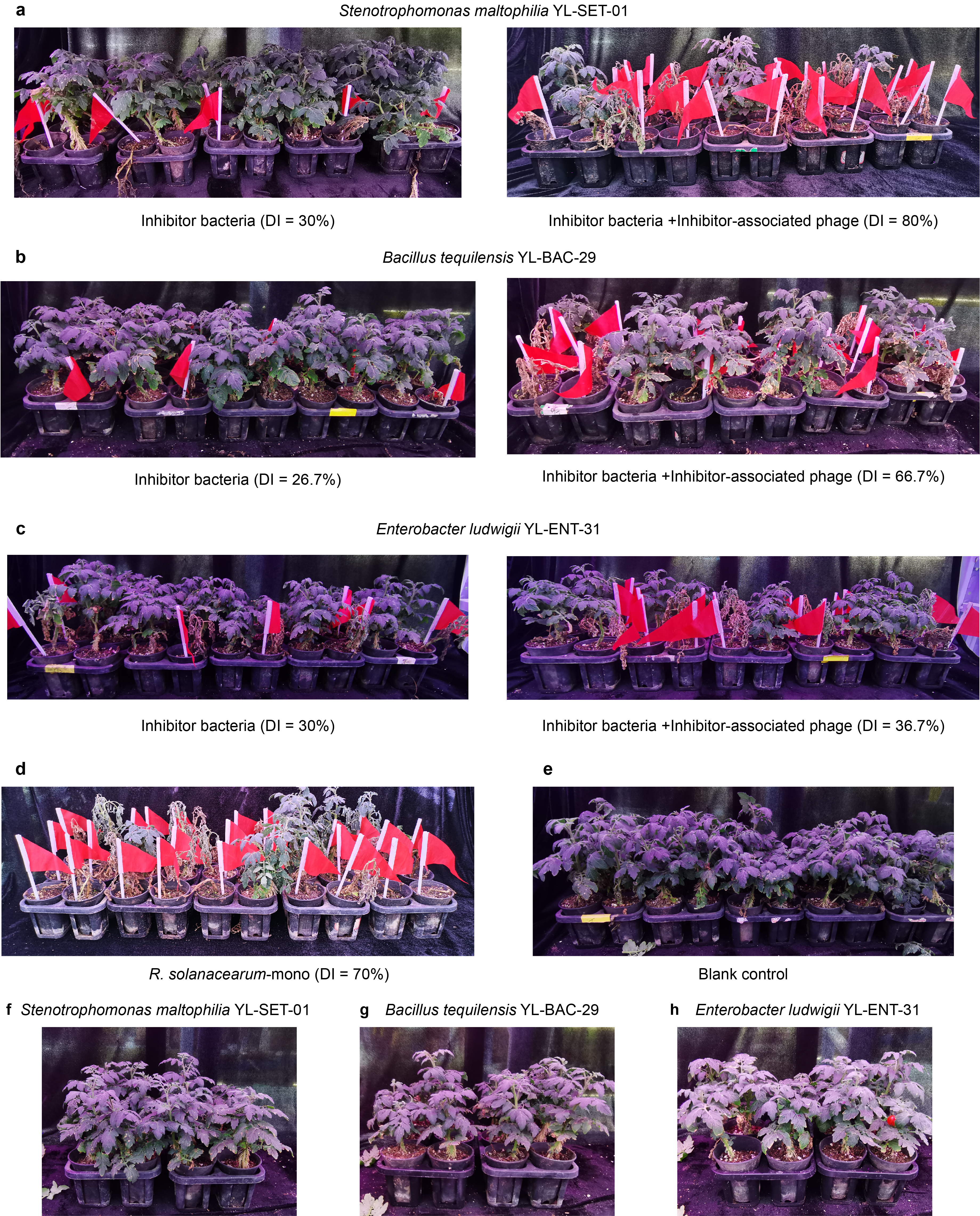


**Supplementary Figure 9. Photograph of tomato plants at the end of greenhouse experiment. a-c**: Treatments with plants inoculated with *R. solanacearum* and different ‘inhibitor bacteria’ in the presence (right) and absence (left) of ‘inhibitor-associated phages’. **d**: ‘*R. solanacearum*-only’ control plants inoculated only with *R. solanacearum*. **e**: Blank control inoculated only with sterile water without any bacteria or phages. **f-h**: Treatments with plants inoculated with different ‘inhibitor bacteria’ only. In panels **a**-**d**, DI represents average disease incidence and diseased plants are highlighted with red flags. No disease symptoms were observed in **e-h**.

**Supplementary Figure 10**


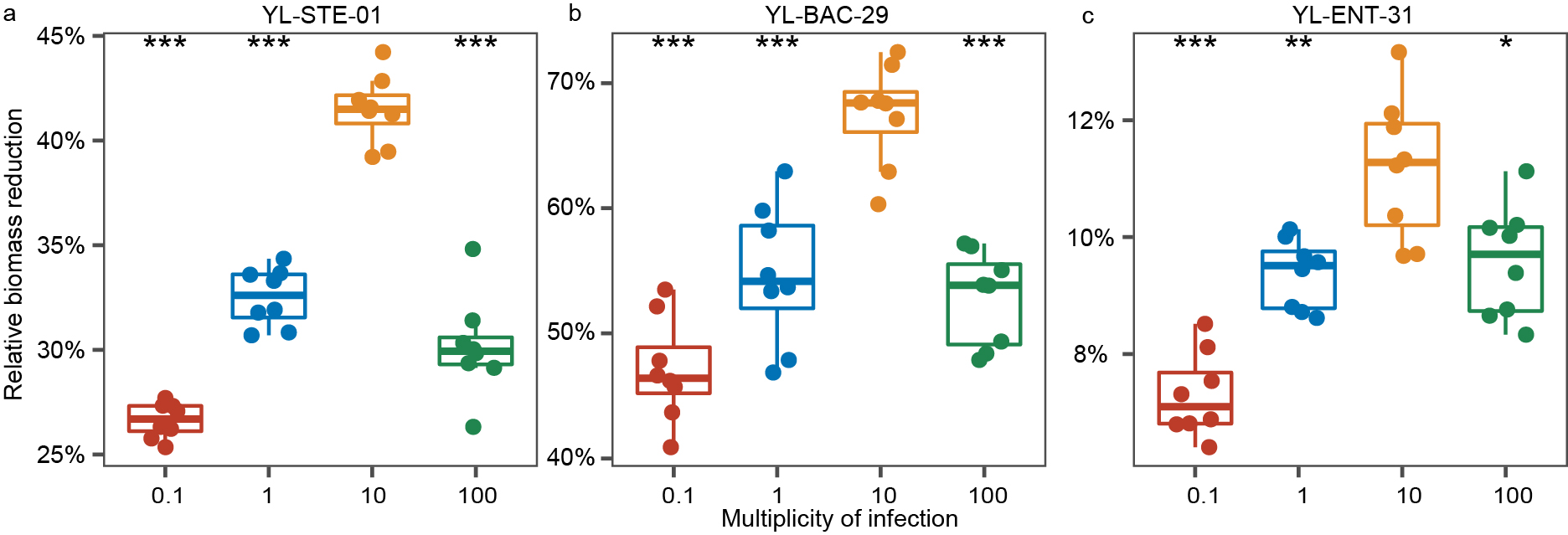


**Supplementary Figure 10. Comparison of the relative bacterial biomass reduction at different initial multiplicity of infections (M.O.I.).** With all‘inhibitor-associated phages’, highest bacterial biomass reduction was observed with M.O.I = 10. Statistical significance between treatments was determined by one-way ANOVA test with *: *P* < 0.05, **: *P* < 0.01, ***: *P* < 0.001, *n* = 8 for all treatments.
